# Balanced X-ray Security Dataset and Enhanced YOLO for Contraband Detection

**DOI:** 10.1038/s41597-025-06322-9

**Published:** 2025-11-26

**Authors:** Songlin Zhang, Dingju Zhu, KaiLeung Yung, Andrew W. H. Ip

**Affiliations:** 1 School of Artificial Intelligence, South China Normal University, Foshan, 528225 China; 2 School of Computer Science, South China Normal University, Guangzhou, 510631 China; 3 Department of Industrial and Systems Engineering, Hong Kong Polytechnic University, HongKong, 999077 China; 4 Department of Mechanical Engineering, University of Saskatchewan, Saskatoon, SK S7N 5A2 Canada

**Keywords:** Research data, Computer science

## Abstract

To address critical challenges in X-ray contraband detection—including severe class imbalance in existing datasets, scarcity of high-quality annotated data, and poor model adaptability to complex scenarios—this study first constructs a balanced X-ray contraband detection dataset. Derived from the SIXray and PIDray datasets, the balanced dataset comprises 13,728 images covering 12 different contraband categories. To resolve class imbalance, a Class-Specific Augmentation Framework (CSAF) with four physical transformations and random undersampling are adopted, ensuring approximately 1,500 samples per category for uniform class distribution. Two improved models (ASEA-Net and CSEC-Net) based on YOLOv11s are proposed for lightweight and high-precision contraband detection tasks. Experiments on the balanced dataset show that ASEA-Net achieves 95.78% accuracy and 93.55% mAP@50, outperforming YOLOv11s by 1.46% and 1.37% respectively with 13.37% fewer parameters; CSEC-Net reduces parameters by 39.91% and FLOPs by 40.38% compared to YOLOv11s, enabling deployment on resource-constrained edge devices. Both models exhibit strong performance in complex scenarios, validating the value of the balanced dataset and the effectiveness of the proposed models for X-ray contraband detection.

## Background & Summary

With the rapid development of public transportation, ensuring the security of luggage has become a vital task in safeguarding public spaces such as airports, train stations, subway stations, and more^1^. Security inspection technology is crucial for safeguarding public lives and property safety, yet achieving rapid and accurate security checks remains a core challenge in the transportation sector. Deep learning methods, leveraging robust computational capabilities and human-like knowledge acquisition mechanisms, have emerged as the dominant paradigm for prohibited item detection.

In security inspection scenarios, such as airports, stations, and other public places, the detection system must be able to accurately identify potentially prohibited items in a very short period of time in order to safeguard public safety and maintain a smooth inspection process. Thus, while accuracy is crucial, the ability to process data in real time and efficiently is equally important^2^. Existing models are becoming increasingly complex, which has driven a corresponding rise in demand for computational resources^3^. This complexity may also affect the models’ real-time responsiveness. Developing efficient and lightweight models that maintain high precision while accommodating the computational constraints of embedded systems is crucial for advancing the practical application of this technology in real-world security scenarios^4^. In practice, the acquisition of X-ray images poses considerable challenges, and the availability of high-quality datasets for security inspection purposes is relatively scarce. This limitation has hindered the advancement of research in this domain. Currently, publicly available X-ray datasets include GDXray^5^, SIXray^6^, OPIXray^7^, CLCXray^8^, and PIDray^9^. The SIXray dataset is a large-scale dataset for prohibited item detection in X-ray security screening. It contains 8,929 X-ray images with manual annotations for prohibited items, primarily covering five object categories: knives, guns, wrenches, pliers, and scissors. The PIDray dataset was collected from various scenarios including airports, subway stations, and train stations, comprising a total of 47,677 images. This dataset was generated using three different security inspection devices, resulting in images of various sizes and color schemes, covering twelve common prohibited item categories: guns, knives, wrenches, pliers, scissors, hammers, handcuffs, batons, spray cans, power banks, lighters, and bullets. Nevertheless, existing datasets and methodologies still exhibit multiple shortcomings. For example, there is a severe class imbalance in contraband samples; this imbalance typically leads models to prioritize the learning of classes with large sample sizes while disregarding those with small sample sizes, ultimately compromising the overall detection performance of the models.

The stacking of prohibited item images and complex image quality issues also constitute formidable challenges in the detection of contraband. Firstly, when multiple objects overlap, X-rays penetrate these objects, resulting in overlapped image contours and a more complex depiction of the image contours. This complexity makes differentiating between objects and accurately extracting boundaries more challenging. Secondly, the placement angles and positions of baggage items are different, leading to varying imaging angles of objects, which drastically change from different perspectives, which increase the detection difficulty and need algorithms to handle objects from various angles and poses. Furthermore, while X-ray imaging easily captures object contour information, it may cause a loss of surface detail features. This loss of detail might lead to difficulties in distinguishing specific features of objects, thereby affecting the accurate detection of prohibited items. Moreover, X-ray imaging cannot directly capture object color information. Instead, it uses measurements of high- and low-energy X-rays to determine an object’s atomic number, distinguishing between organic and inorganic substances and, finally, coloring the object based on the above distinction. So, other features instead of color need to be used to detect prohibited items^10^.

In recent years, research has increasingly focused on leveraging artificial intelligence (AI) to enhance security screening efficiency, an approach demonstrating significant potential for automated contraband detection^11^. Among the most promising AI technical routes is the adoption of convolutional neural networks (CNNs), which have shown notable advantages in identifying and classifying objects within cluttered and overlapping environments^12^. Multiple CNN-based models—including Faster R-CNN, the “You Only Look Once” (YOLO) algorithm, and RetinaNet—have been deployed in X-ray security screening systems, effectively improving detection accuracy while reducing reliance on manual inspection^13^. Despite these advancements, challenges such as object occlusion and data set variability require further optimization to refine detection performance.

The early methods used to detect X-ray prohibited items were primarily based on traditional hand-designed features. In 2011, Baştan *et al*.^14^ investigated the applicability of bag of visual words (BoW) methods to the classification and retrieval of X-ray images. Turcsany *et al*.^15^ proposed a novel Bag-of-Words (BoW) representation scheme for image classification tasks. In 2016, Kundegorski *et al*.^16^ investigated the relative performance of different feature descriptors in the Bag of Visual Words (BoVW) model. Traditional handcrafted feature-based recognition methods exhibit high interpretability in object identification processes. However, they suffer from limitations such as the inability of manually engineered features to sufficiently capture complex object characteristics and variations, coupled with poor generalization capability when handling complex scenarios or novel object categories.

Driven by the rapid advancement and demonstrated potential of deep learning, the predominant focus of research has migrated toward deep neural network-based methodologies for object recognition. In 2016, Akçay *et al*.^17^ first introduced convolutional neural networks (CNNs) to the field of X-ray contraband detection. By utilizing pre-trained CNN models for automatic classification of prohibited items in X-ray images, they significantly enhanced detection accuracy. Wei *et al*.^7^ introduced the De-occlusion Attention Module (DOAM), which synthesizes attention maps through edge features and material characteristics. Viriyasaranon *et al*.^18^ proposed the MFAnet, which employs a Multi-scale Dilated Convolution module (MDConv) to dynamically aggregate convolutional features with varying dilation rates, combined with a Fusion Feature Pyramid Network (FusionFPN) to mitigate aliasing effects in cross-scale feature fusion, significantly enhancing the localization performance for small-scale targets. Wang *et al*.^19^ incorporated Transformer layers into the backbone network of YOLOv5 and adopted global attention mechanisms along with an adaptive spatial feature fusion algorithm, significantly enhancing the model’s precision. The algorithm achieves a mean Average Precision (mAP) of 91% on the OPIXray dataset. Li *et al*.^20^ proposed the SC-YOLOv8 model, which incorporates an improved C2F_DCN module utilizing deformable convolutions to adaptively adjust the position and shape of the receptive field, thereby accommodating target diversity. Jia *et al*.^21^ proposed ForkNet, whose core lies in a foreground-background specific feature learning strategy designed to decouple feature entanglement caused by overlapping objects. To enhance feature discriminability, ForkNet extracts foreground and background features via different backbones. Its core design involves the application of feature disentanglement, which specifically targets redundant information in foreground features to remove such noise and optimize feature representation. Zhu *et al*.^22^ proposed a frequency-aware dual-stream transformer network (FDTNet), which incorporates two dedicated branches. These branches independently handle RGB features and frequency-domain characteristics, and further achieve effective feature fusion by leveraging global attention and channel-wise attention mechanisms. In 2025, Zhu *et al*.^23^ propose an Adaptive and Efficient Focus Network (AEFNet) that enhances automated contraband detection by leveraging targeted region localization. Chen *et al*.^24^ propose the Receptive Field Large Separable Kernel Attention (RFLSKA) module, enabling adaptive spatial receptive field adjustment for multi-scale context aggregation. Complementarily, the Wise-SIoU loss function is designed to suppress false alarms and omission errors in contraband detection, significantly boosting model robustness across diverse scenarios. Current research confirms that dynamic feature selection, attention mechanisms, and customized loss functions significantly mitigate false alarms caused by overlapping objects and cluttered backgrounds in X-ray security imagery. These innovations deliver robust technical foundations for deploying reliable inspection systems in high-traffic public facilities.

While the application of deep neural networks has enhanced the performance of object detection algorithms, it inevitably introduces significant computational overhead. Security inspection, as a process that must be completed within strict time constraints—particularly in high-traffic scenarios such as subways or airports—demands rapid and efficient procedures to maintain operational flow and user experience. Consequently, research on lightweight object detection models has gained momentum alongside advancements in detection technologies. Ren *et al*.^25^ proposed a lightweight object detection framework based on the YOLOv4 algorithm. By incorporating a Lightweight Feature Pyramid Network (LFPN) and a Convolutional Block Attention Module (CBAM), the approach effectively achieves multi-scale feature fusion, reducing the model’s floating point operations (FLOPs) and parameter count (Params) to 1/5 and 1/3 of the original values respectively. Liu *et al*.^26^ adopted MobileNetV3 as the backbone network for YOLOv4 and applied depthwise separable convolutions to optimize its neck and head structures. They designed an Adaptive Spatial-Channel Attention module to enhance the neck architecture of YOLOv4. Zhang *et al*.^27^ proposed an improved YOLOv7-tiny model that employs a lightweight network called FasterNet as the feature extraction backbone. Within the neck layer, they replaced the ELAN module with a New-ELAN module incorporating Partial Convolution (PConv). The refined model achieves overall lightweighting while maintaining competitive detection accuracy. Zhou *et al*.^28^ proposed Fine-YOLO, introducing a Low-Parameter High-Density Feature Integration module. This module enhances small target detection in high-level visual features while maintaining model lightweighting. Despite rapid advancements in artificial intelligence and object detection technologies, significant opportunities continue to exist for enhancing efficiency and precision within the security screening domain. Major technical challenges remain in simultaneously implementing lightweight designs while maintaining detection accuracy.

Based on the above context, to address these limitations and shortcomings in the existing research, our research is motivated by the pursuit of a lightweight model capable of efficient and accurate detection of contraband in X-ray images. Therefore, we propose ASEA-Net, an improved model based on YOLOv11s, which achieves enhanced detection capabilities while ensuring a more lightweight architecture. The contributions of this paper are as follows:

1. A balanced X-ray contraband detection dataset containing 13,728 images has been constructed based on the SIXray and PIDray datasets.
2. ASEA-Net: The introduction of EfficientNet_B0 to replace YOLOv11s’ backbone network enables model compression while preserving detection speed and accuracy. Replacing traditional convolutions in the model head architecture with the ADown downsampling module not only makes the model more lightweight but also enhances its capability to detect overlapping and occluded prohibited items. The lightweight detection head SEAMHead is introduced by replacing the original Detect head with the improved SEAMHead, which integrates the Separated and Enhancement Attention Module (SEAM) to enhance the model’s ability to handle occluded targets. The AFGCAttention mechanism is introduced in the neck of the model to enhance fine-grained interaction between global and local information, thereby improving the model’s discriminative capabilities in complex scenes.
3. We further propose an even more lightweight model, CSEC-Net: Building upon the EfficientNet_B0 backbone network and the SEAMHead detection head, CSEC-Net incorporates a Coordinate Attention-based High-level Screening Feature Pyramid Module (CA-HSFPN) that significantly reduces the model’s parameters and computational burden. Additionally, the introduction of a C3k2-EMA module in the model’s neck enhances the network’s ability to process multi-scale features.

### Overview of the YOLOv11 model

The YOLOv11 model introduces a series of innovative designs within the YOLO series to enhance object detection accuracy and computational efficiency. Its architecture comprises three core functional modules: the Backbone, the Neck, and the Head. The Backbone adopts C3k2 modules, optimizing feature extraction through the use of small convolutional kernels and feature map partitioning. This approach reduces computational complexity and improves efficiency. Simultaneously, the model incorporates a C2PSA module to enhance spatial attention mechanisms, enabling the model to dynamically prioritize critical image regions requiring high-resolution analysis.

**Fig. 1:**
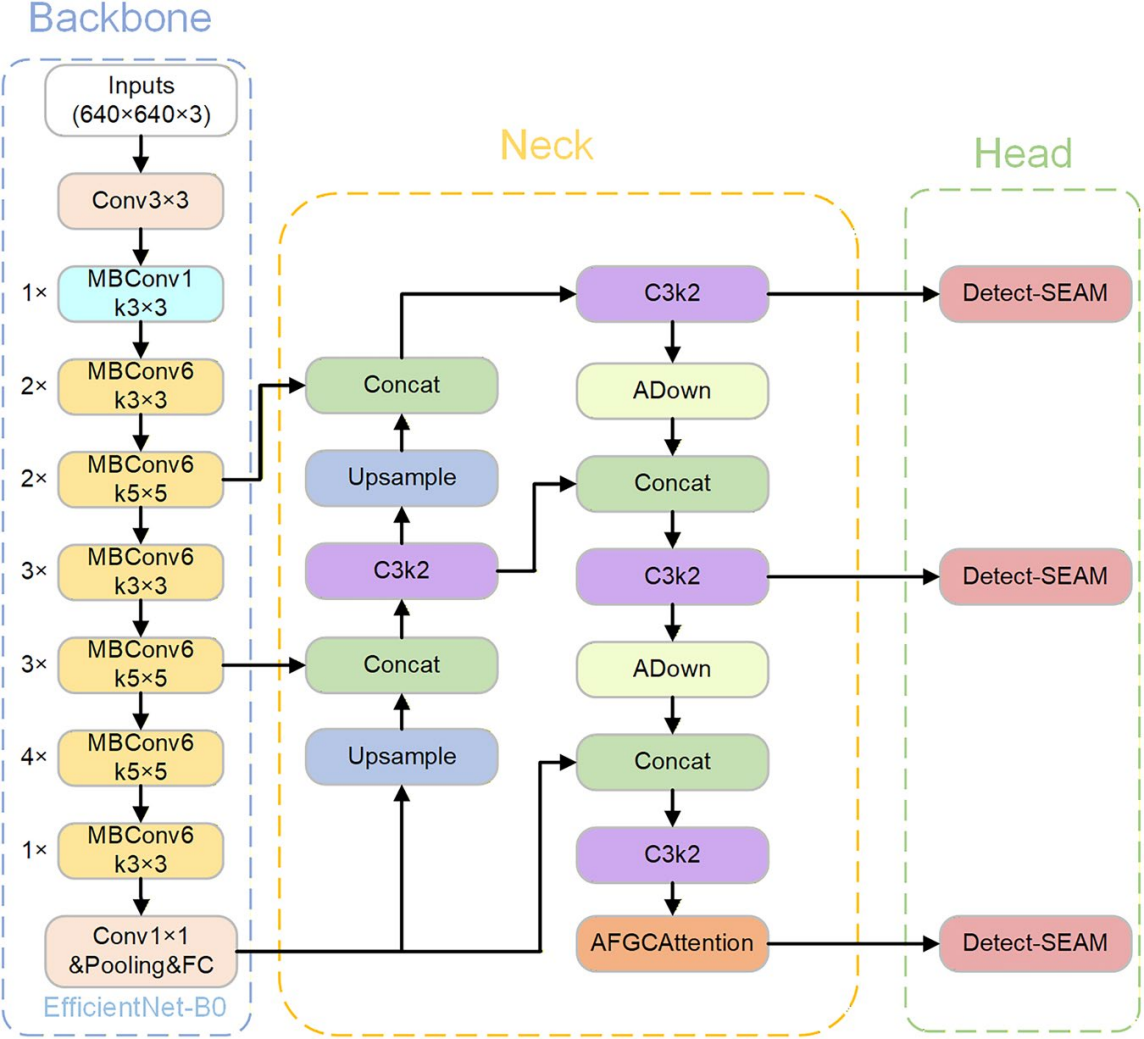
The network architecture of ASEA-Net.

**Fig. 2:**
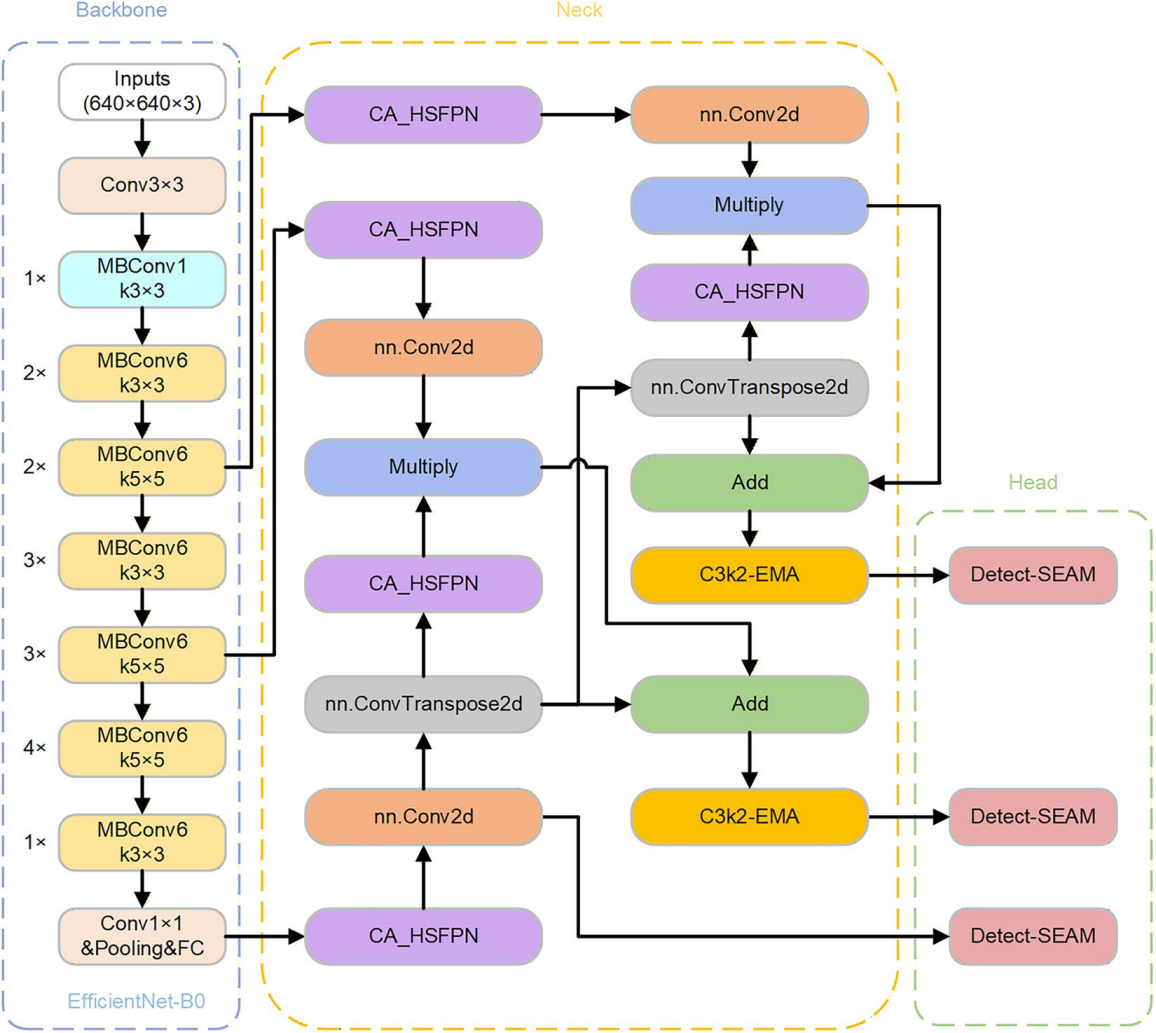
The network architecture of CSEC-Net.

Within the YOLOv11 series, this study employs the compact variant YOLOv11s. Though less accurate than larger-scale models, it maintains high detection efficiency in resource-constrained environments, demonstrating well-balanced performance for real-time detection tasks.By integrating four innovative modules into YOLOv11, ASEA-Net fundamentally reduces parameter count at the architectural level while enhancing feature extraction capability, computational efficiency, and contraband detection performance. The overall architecture of ASEA-Net is shown in Fig. 1. Simultaneously, while retaining the EfficientNet backbone network and the SEAMHead detection architecture, we propose another more lightweight model named CSEC-Net by integrating the Exponential Moving Average (EMA) attention mechanism into the C3k2 module and adopting a Cross-stage Aggregation Hierarchical Feature Pyramid Network (CA-HSFPN) to replace the traditional Feature Pyramid. CSEC-Net exhibits a minor improvement in detection accuracy but achieves a significant reduction in the number of parameters, enabling deployment on more resource-constrained edge devices. The overall architecture of CSEC-Net is shown in Fig. 2.

### Lightweight backbone extraction network

Although YOLOv11s demonstrates outstanding performance, its backbone network still suffers from large parameter size and high computational complexity compared to mainstream lightweight networks. This hinders rapid and effective detection of contraband items in complex environments. To address this limitation, we replace the backbone of YOLOv11s with EfficientNet-B0.

EfficientNet^29^ is an efficient convolutional neural network architecture proposed by Google Research in 2019. The model series (B0 to B7) balances accuracy and computational efficiency: B0 serves as the baseline model, with subsequent versions increasing model scale and computational demands while improving accuracy. EfficientNet-B0 employs a novel compound scaling mechanism—traditional scaling methods typically increase only network depth, width, or input resolution, whereas compound scaling enables simultaneous scaling across depth, width, and resolution dimensions. This allows the network to more efficiently utilize additional computational resources, governed by a fixed coefficient applied uniformly to the entire model. Specifically, the EfficientNet-B0 model consists of multiple stacked convolutional layers. Each layer contains convolutional modules based on the Mobile Inverted Bottleneck Convolution (MBConv) structure to extract multi-level features. Additionally, the model incorporates Swish activation functions to enhance computational efficiency and integrates Squeeze-and-Excitation (SE) modules to strengthen feature channel attention mechanisms. The baseline network structure of EfficientNet-B0 is detailed in Table 1^29^.

**Table 1:** EfficientNet-B0 baseline network.

Stage	Operator	Resolution	Channels	Layers
1	Conv3x3	224 × 224	32	1
2	MBConv1,k3x3	112 × 112	16	1
3	MBConv6,k3x3	112 × 112	24	2
4	MBConv6,k5x5	56 × 56	40	2
5	MBConv6,k3x3	28 × 28	80	3
6	MBConv6,k5x5	14 × 14	112	3
7	MBConv6,k5x5	14 × 14	192	4
8	MBConv6,k3x3	7 × 7	320	1
9	Conv1x1&Pooling&FC	7 × 7	1280	1

**Fig. 3:**
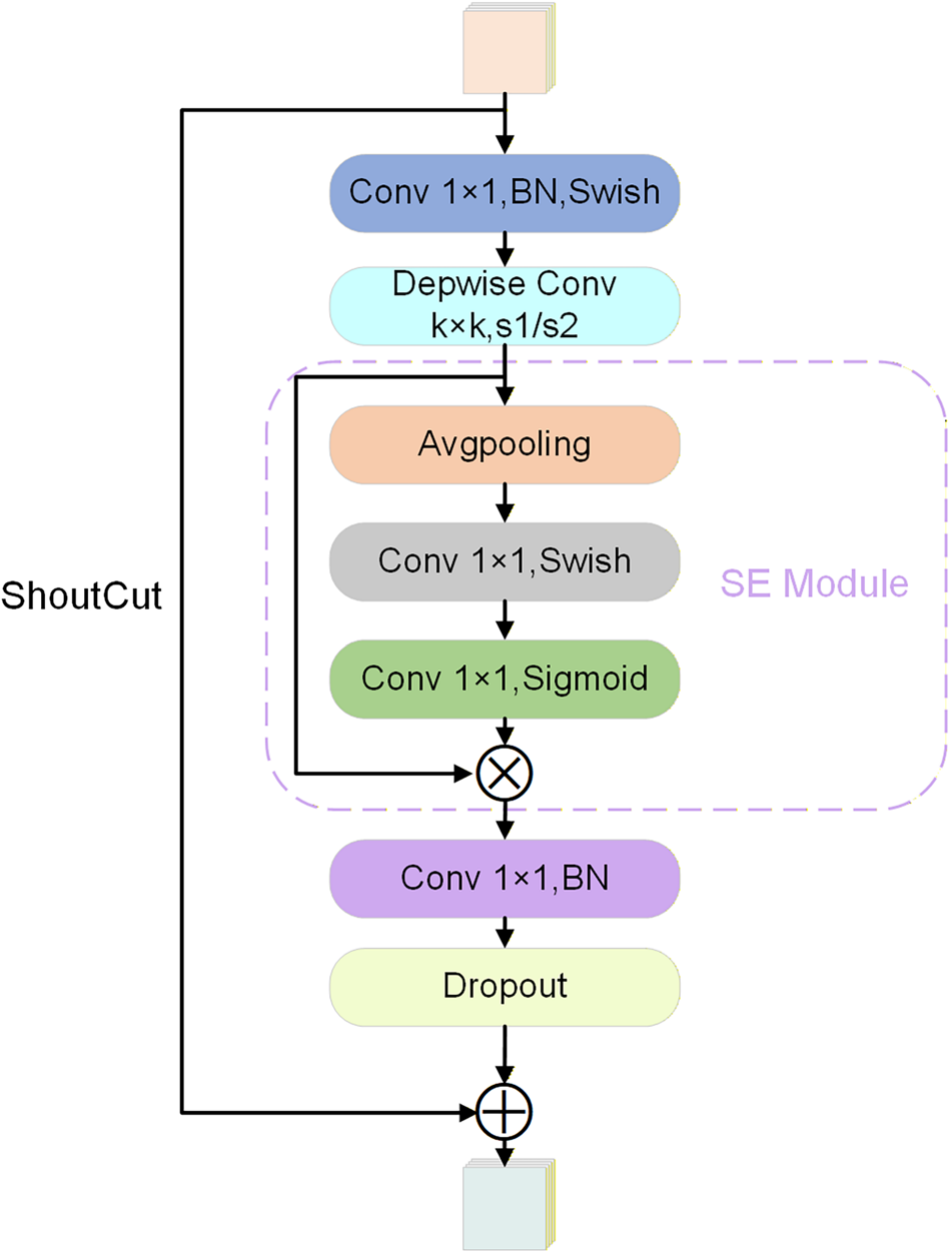
MBConv structure diagram.

**Fig. 4:**
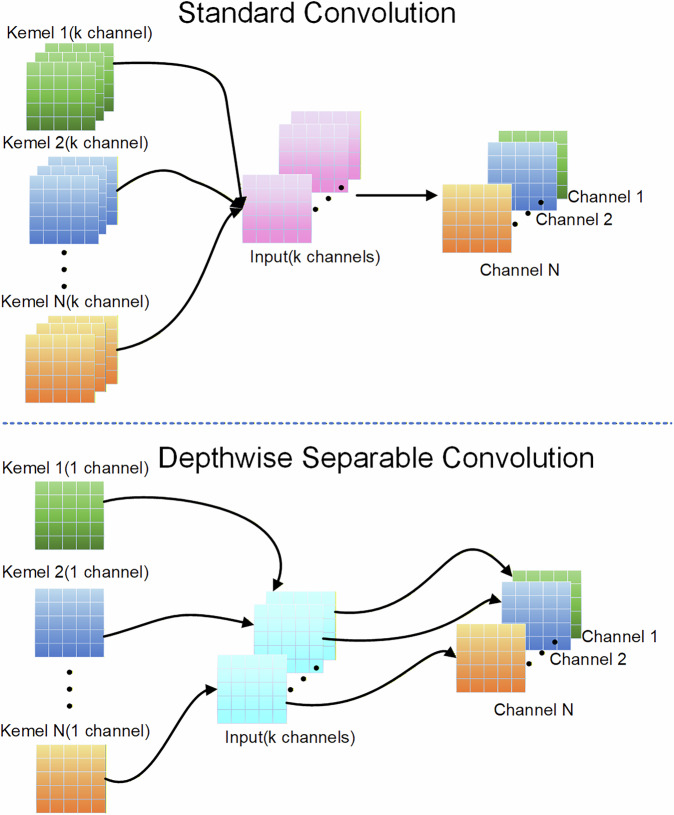
Comparison of the processes of standard convolution and depthwise separable convolution.

The MBConv design used in EfficientNet (shown in Fig. 3) combines depthwise separable convolution and dilated convolution to form a lightweight yet highly efficient neural network structure. This approach integrates the concepts of inverted residuals and bottlenecks to enhance model efficiency and performance. Fundamentally, the MBConv module consists of three key components: depthwise separable convolution, projection convolution, and batch normalization. Depthwise separable convolution operates through two distinct stages: depthwise convolution followed by pointwise convolution. During the first stage, spatial convolution is applied separately to each input channel. The subsequent pointwise convolution then reduces computational load and parameter count. This sequential process enables efficient feature extraction while dramatically cutting computational overhead. Projection convolution adjusts channel dimensions between input and output feature maps through pointwise convolution, ensuring compatibility between layers. As illustrated in Fig. 4 comparing conventional convolution versus depthwise separable convolution, batch normalization critically mitigates internal covariate shifts to accelerate convergence during optimization while serving as a regularization mechanism against overfitting. The synergistic operation of these three elements significantly enhances the MBConv architecture’s parameter efficiency and computational performance, enabling exceptional capability in lightweight models and boosting overall system efficiency^30^.

### ADown module

In complex security inspection scenarios, object occlusion and overlap often lead to missed detection of prohibited items. Traditional convolution with large downsampling strides may cause feature loss when obscured prohibited items exhibit weak characteristics. To address detection challenges in intricate environments and enhance accuracy for overlapping prohibited items, this study replaces conventional convolution in the YOLOv11 head structure with the ADown^31^ downsampling module.

**Fig. 5:**
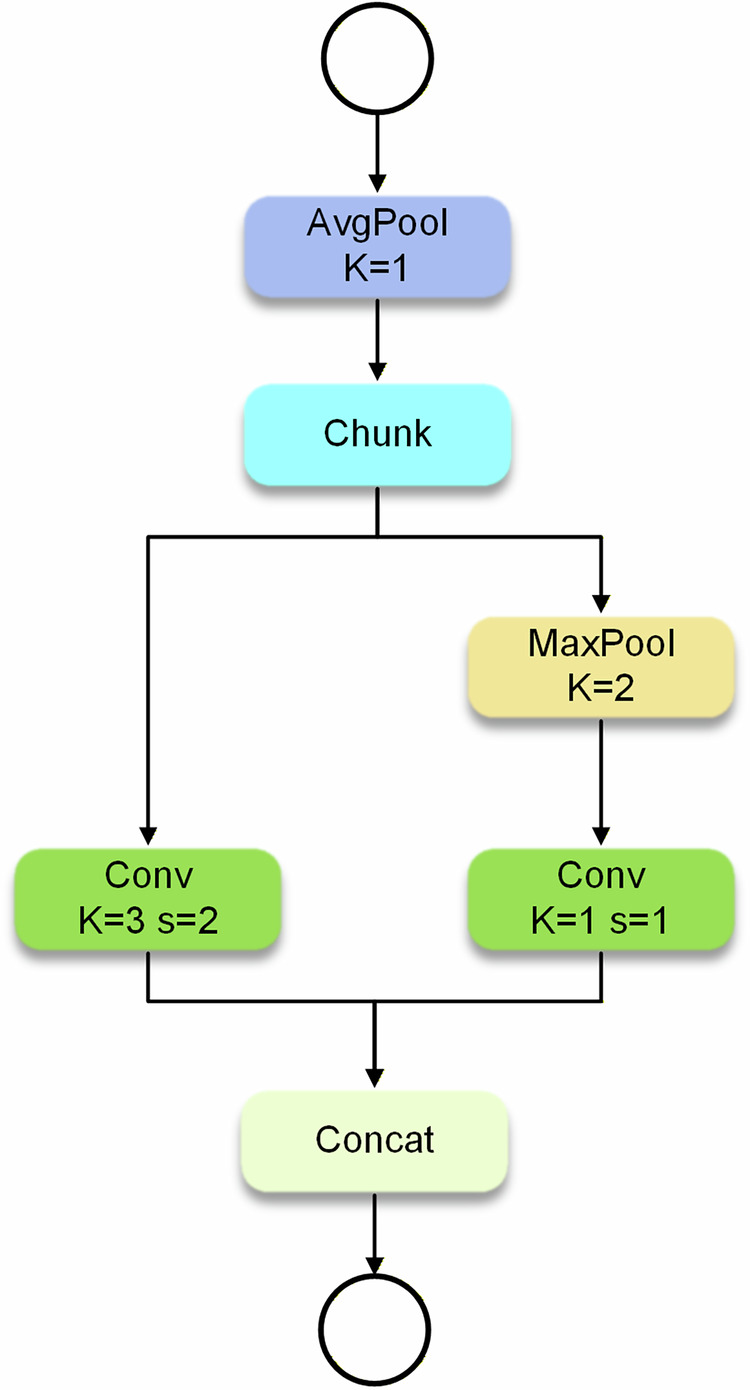
Adown network structure.

ADown integrates average pooling and max pooling techniques: average pooling preserves global information, while max pooling captures significant local features. Their combination enables multi-scale feature extraction. Compared to traditional convolution, ADown reduces spatial dimensions by adjusting convolutional stride lengths, retaining more image information while decreasing spatial resolution. Crucially, this module possesses adaptive learning capabilities tailored to different detection scenarios. Furthermore, ADown reduces model complexity by decreasing parameter counts, enhancing detection accuracy while maintaining model lightweightness. The model structure of the ADown module is shown in Fig. 5. This structure first employs average pooling (pooling kernel K = 1) to smooth the input feature map. Subsequently, the input is directed into two parallel paths: The first path applies a convolutional operation (convolution kernel K = 3, stride s = 2), simultaneously performing convolution and downsampling. The second path first reduces spatial resolution through max pooling (pooling kernel K = 2), and then employs a convolutional operation (convolution kernel K = 1, stride s = 1) to extract key features without altering the spatial dimension. The processed outputs from the two branches are fused via concatenation, ultimately generating a composite feature map that incorporates both spatial information and essential feature details. This approach allows the model to capture finer details from the input, which is crucial for contraband detection tasks—since the target objects in such tasks are typically small and irregularly shaped. By improving feature extraction efficiency and preserving more spatial context information, the ADown module enhances the model’s ability to distinguish contraband from normal items in complex environments, thereby increasing detection accuracy while maintaining computational efficiency. These improvements make the model more robust in real-time contraband detection tasks within security inspection systems.

### SEAMHead

**Fig. 6:**
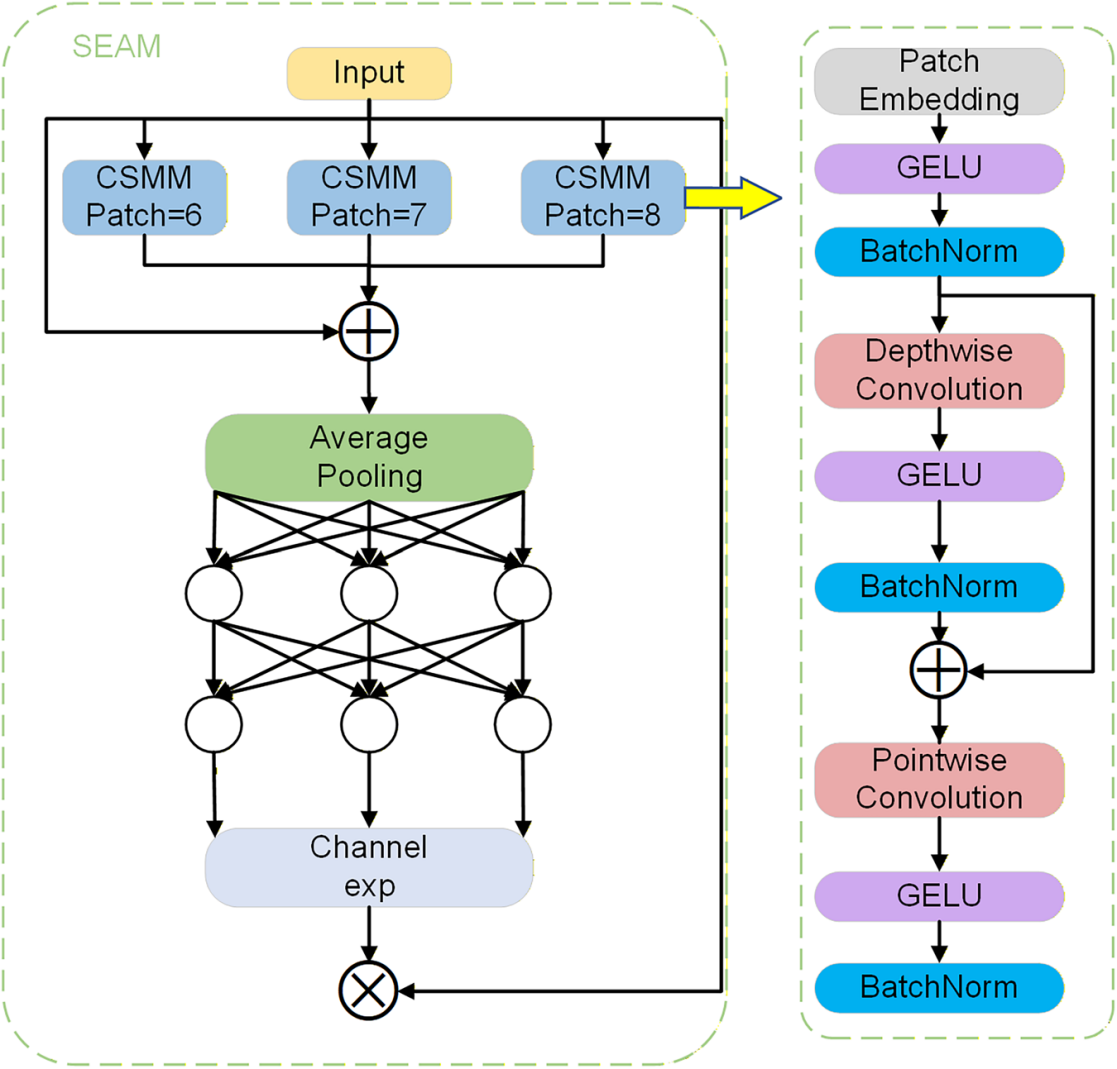
SEAM attention mechanism network structure.

To address the issues of feature misalignment, local aliasing, and critical feature loss due to occlusion, Yu *et al*.^32^ proposed the Separated and Enhancement Attention Module (SEAM)—a multi-head attention network designed to enhance multi-scale detection performance by amplifying key image regions while suppressing background interference. As shown in Fig. 6, the left side illustrates the overall architecture of SEAM, while the right side details the structure of the Channel-Spatial Mixed Module (CSMM). The CSMM extracts multi-scale features from different image regions and employs depthwise separable convolution to learn the correlation between spatial and channel dimensions.

SEAM first utilizes depthwise separable convolution with residual connections to perform convolution operations separately on each channel. This approach reduces parameter count while learning the importance of individual channels. However, it overlooks inter-channel dependencies. To address this limitation, SEAM integrates pointwise convolution (1 × 1 convolution) to combine outputs from different depthwise convolutions. Additionally, a two-layer fully connected network fuses cross-channel information, enhancing inter-channel interactions. SEAM simultaneously captures long-range dependencies in both spatial and channel dimensions, enabling it to model relationships between occluded and non-occluded objects, thereby mitigating the negative impact of occlusion.

The output logits of the fully connected layer undergo an exponential transformation, mapping values from [0, 1] to [1, e]. This monotonic normalization enhances the model’s tolerance to positional deviations. Finally, SEAM’s output serves as attention weights, multiplied with the original feature map to boost the detection capability of the detection head and improve occlusion robustness^33^.

By integrating SEAM into the detection head of YOLOv11s, the model strengthens associations between occluded and non-occluded regions, reducing occlusion-induced accuracy degradation. Furthermore, SEAM improves inference speed without significantly increasing computational costs and enhances model robustness in complex environments.

### AFGCAttention module

**Fig. 7:**
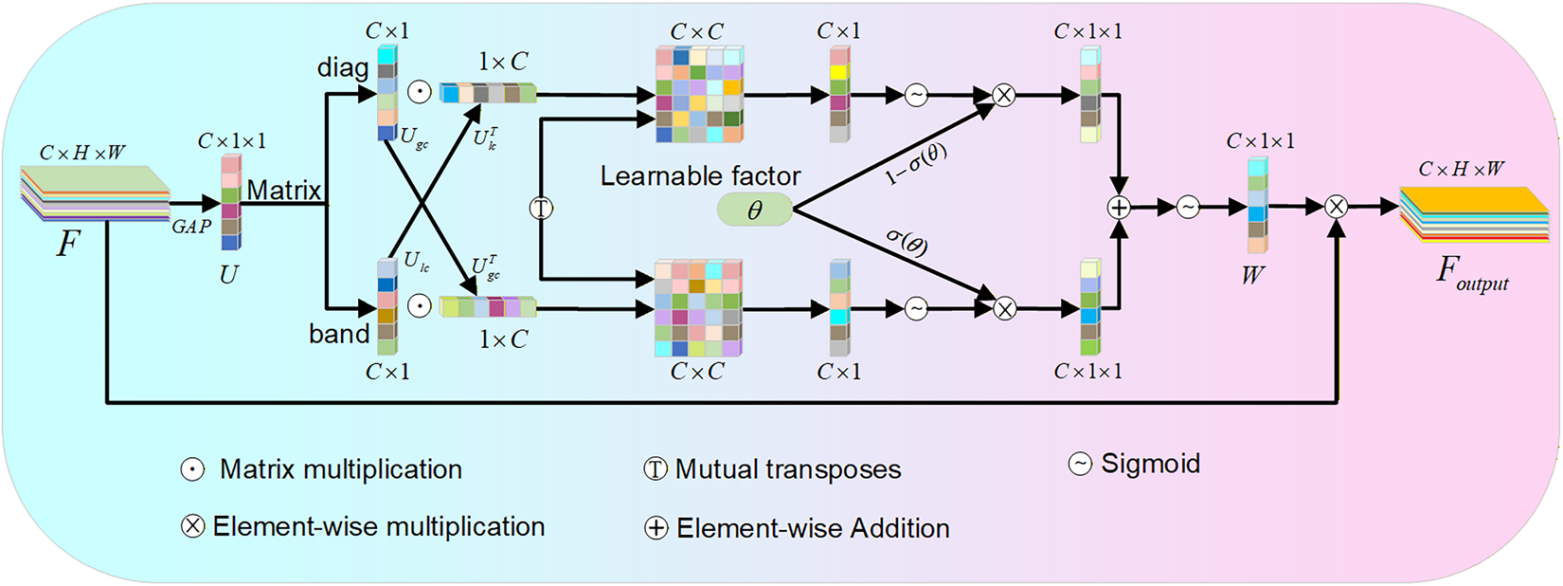
AFGCAttention module.

To address the challenges of complex backgrounds and stacked items in contraband detection, this paper introduces the AFGCAttention^34^ (Adaptive Fine-Grained Channel Attention) mechanism. This attention mechanism dynamically adjusts the weights of each channel in the feature map based on its importance, thereby optimizing feature selection. By enhancing the fine-grained interaction between global and local information, AFGCAttention allows the model to focus more effectively on features relevant to subsequent tasks while suppressing insignificant or irrelevant background noise. As shown in Fig. 7, this capability significantly improves the model’s discriminative power in complex scenarios.

The n-th channel element of $U$ can be represented as the channel descriptor $U\in {R}^{C}$ generated by global average pooling on the given feature map $F\in {R}^{C\times H\times W}$.

1 $${U}_{n}={GAP}\left({F}_{n}\right)$$

where: ${F}_{n}$ represents the pixel values of the n-th channel feature map, and ${GAP}(X)$ is the global average pooling function. Through this function, the feature map transforms from the shape of $C\times H\times W$ to $C\times 1\times 1$.

We use interval matrix $B$ to capture local inter-channel interactions while minimizing model parameters and set $B=[{b}_{1,}{b}_{2},{b}_{3},\ldots ,{b}_{k}]$ as follows:

2 $${U}_{{lc}}=\mathop{\sum }\limits_{i=1}^{k}U\cdot {b}_{i}$$

where: $U$ represents the channel descriptor, ${U}_{{lc}}$ represents the local information, and $k$ represents the number of adjacent channels.

Using Conv1D for implementation, we incorporate a diagonal matrix $D=[{d}_{1,}{d}_{2},{d}_{3},\ldots ,{d}_{c}]$ to capture global channel information, enhancing inter-channel dependency modeling.

3 $${U}_{{gc}}=\mathop{\sum }\limits_{i=1}^{c}U\;\cdot \;{d}_{i}$$

where: ${U}_{{gc}}$ represents the global information, and $C$ represents the number of channels.

Employing Conv2D as the computational backbone, we unify globally contextual features (diagonal matrix) and locally structured features (band matrix)^35^. Their inter-granular dependencies are explicitly learned through a correlation matrix $M$.

4 $$M={U}_{{gc}}\cdot {U}_{{lc}}^{T}$$

where: $M$ represents the correlation matrix.

Next, to accurately allocate feature weights and reduce computational complexity, we introduce an adaptive fusion strategy. We extract row and column information from the correlation matrix and its transpose as the weight vectors for global and local information. Dynamic fusion is achieved through a learnable factor, as shown below:

5 $${U}_{{gc}}^{w}=\mathop{\sum }\limits_{j}^{c}{M}_{i,c},\,i\in 1,2,3\ldots c$$

6 $${U}_{{lc}}^{w}=\mathop{\sum }\limits_{j}^{c}{\left({U}_{{lc}}\cdot {U}_{{gc}}^{T}\right)}_{i,j}=\mathop{\sum }\limits_{j}^{c}{M}_{i,j}^{T},\,i\in 1,2,3\ldots c$$

7 $$W=\sigma \left(\sigma \left(\theta \right)\times \sigma \left({U}_{{gc}}^{w}\right)+\left(1-\sigma \left(\theta \right)\right)\times \sigma \left({U}_{{lc}}^{w}\right)\right)$$

In which ${U}_{{gc}}^{w}$ and ${U}_{{lc}}^{w}$ are the fused global and local channel weights, and $c$ is the number of channels. This parameter represents the s-type activation function. The strategy avoids redundant interactions between global and local information while further promoting their collaboration. Ultimately, the model is able to selectively emphasize key information and suppress irrelevant features, thereby achieving precise weight allocation for the relevant features. Finally, the obtained weights are element-wise multiplied with the input feature map to obtain the final output feature map.

8 $${F}_{{output}}=F\otimes W$$

where: $F$ is the input feature map, ${F}_{{output}}$ is the output feature map, and $\otimes$ represents the element-wise multiplication operation.

### Improve C3k2 module

**Fig. 8:**
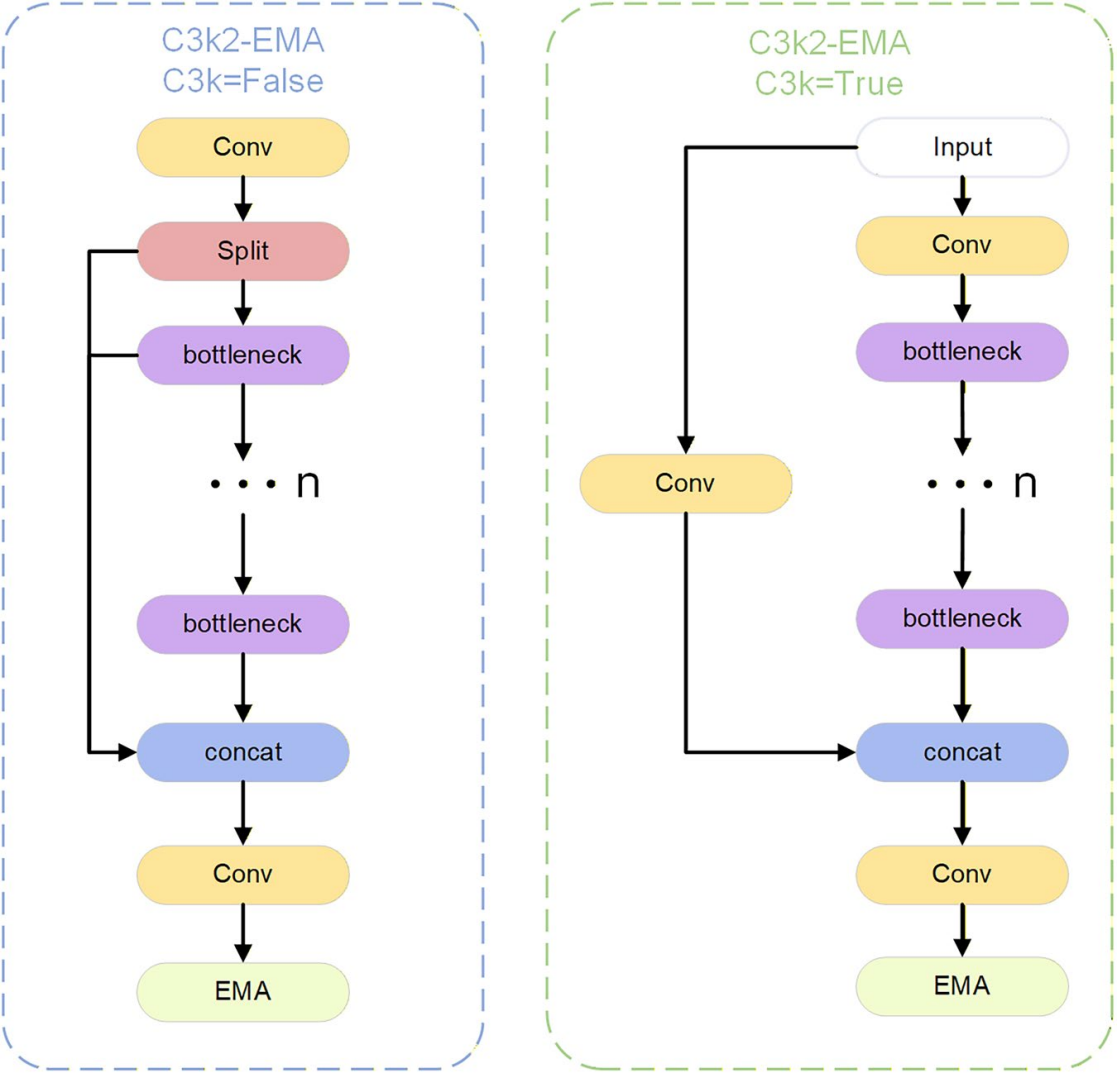
C3k2-EMA structure.

In YOLOv11, the C3k2 module is utilized for feature extraction. When detecting contraband, variations in the shape, size, and texture characteristics of these items can lead to differences in image quality, ultimately contributing to improved model performance. By integrating the EMA (Efficient Multi-Scale Attention) mechanism into the C3k2 module, the C3k2-EMA module is formed, significantly enhancing the network model’s multi-scale feature processing capabilities. The structural diagram is illustrated in the Fig. 8.

**Fig. 9:**
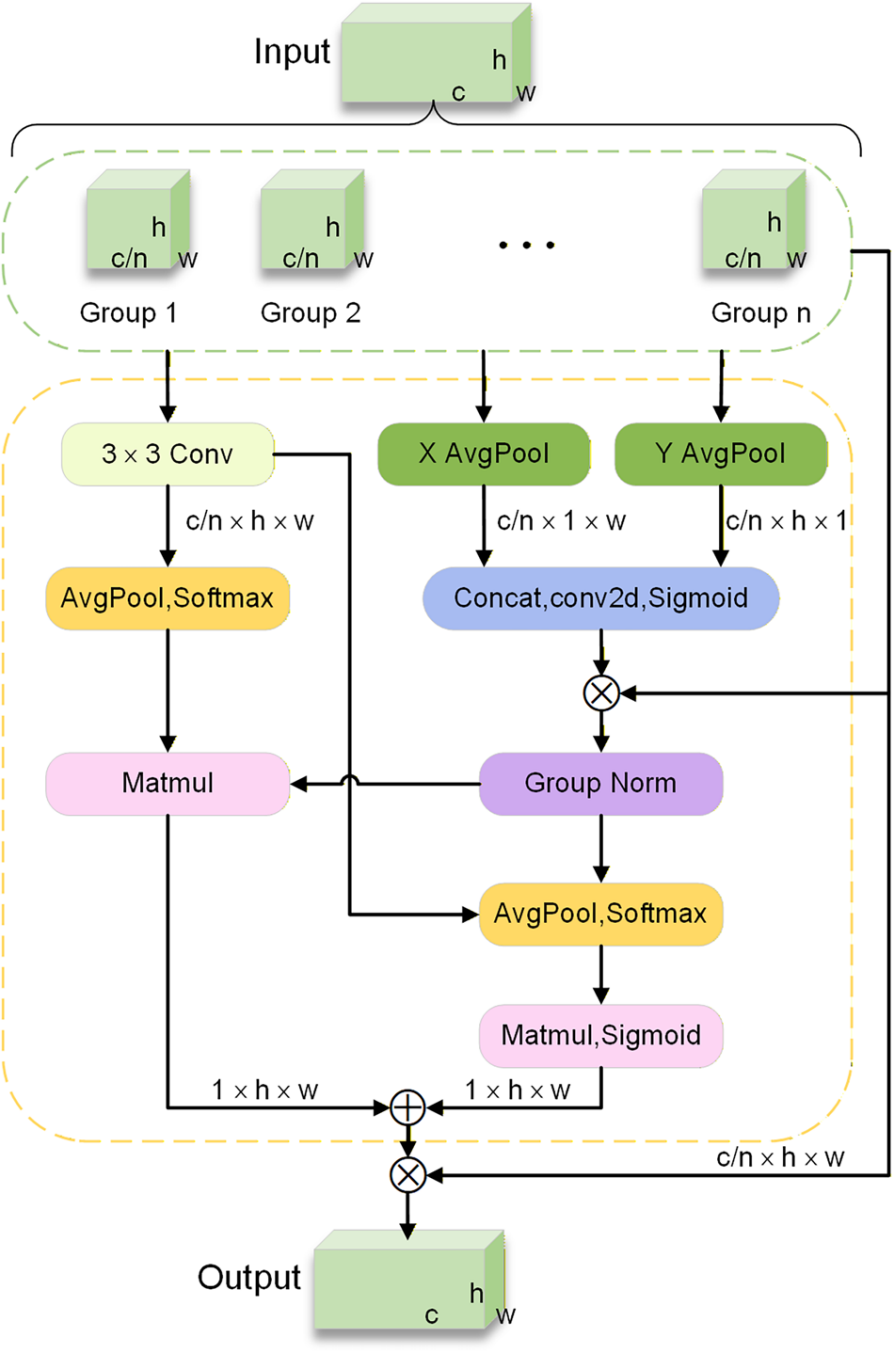
EMA attention mechanism structure.

Attention mechanisms are frequently employed in object detection tasks to identify and process critical information. This approach significantly enhances the model’s sensitivity to contraband, thereby enabling the extraction of more comprehensive feature information. Consequently, the EMA attention mechanism is introduced to enhance the information fusion capability between channels through feature grouping, parallel sub-networks, and cross-spatial learning methods. By preserving channel information, specific channel dimensions are converted into batch dimensions to enable cross-dimensional interaction within sub-networks. Ultimately, the outputs of these parallel networks are combined to enhance the information fusion capability among channels. The structural diagram of the EMA attention mechanism is illustrated in Fig. 9.

The EMA attention mechanism enhances the CA attention mechanism by decomposing the channel into two one-dimensional feature encodings. Furthermore, it includes a parallel branch that employs a convolutional kernel size of 3 × 3.

For the given feature $X\in {R}^{c\times h\times w}$, group feature grouping is utilized to partition the feature dimension into n sub-features. In the case of a 1 × 1 convolution branch, the input feature map undergoes one-dimensional global average pooling across the two spatial dimensions, followed by concatenation, activation, and multiplication operations facilitate the exchange of information across dimensions. For the 3 × 3 branch, we extract additional multi-scale information from the input feature map using a 3 × 3 convolution. Subsequently, cross-spatial learning is employed to perform two-dimensional average pooling and maximum pooling operations on the 3 × 3 branch. This process yields dimensional representations of ${R}_{3}^{c/n\times 1\times 1}$ and ${R}_{3}^{c/n\times h\times w}$, respectively.

The feature channel output encoded by the 1 × 1 branch after passing through group normalization is denoted as ${R}_{1}^{c/n\times h\times w}$, while the unified feature dimension is represented as ${R}_{1}^{c/n\times h\times w}\times {R}_{3}^{c/n\times 1\times 1}$. Conversely, the feature channel output encoded by the 1 × 1 branch following group normalization can be expressed as ${R}_{1}^{c/n\times 1\times 1}$, and the unified feature dimension after aggregation is given by ${R}_{1}^{c/n\times 1\times 1}$ × ${R}_{3}^{c/n\times h\times w}$.

Finally, the matrices after dimension unification are multiplied to fuse the output feature information of each group and obtain two spatial attention weight values; after applying the sigmoid activation function, the final feature map is generated, which preserves spatial information without increasing model complexity^36^. The formula for the two-dimensional global pooling operation is presented in Eq. (9):

9 $${Z}_{c}=\frac{1}{h\times w}\mathop{\sum }\limits_{j}^{h}\mathop{\sum }\limits_{i}^{w}{x}_{c}\left(i,j\right)$$

### Improvement of CA-HSFPN module-based neck networks

**Fig. 10:**
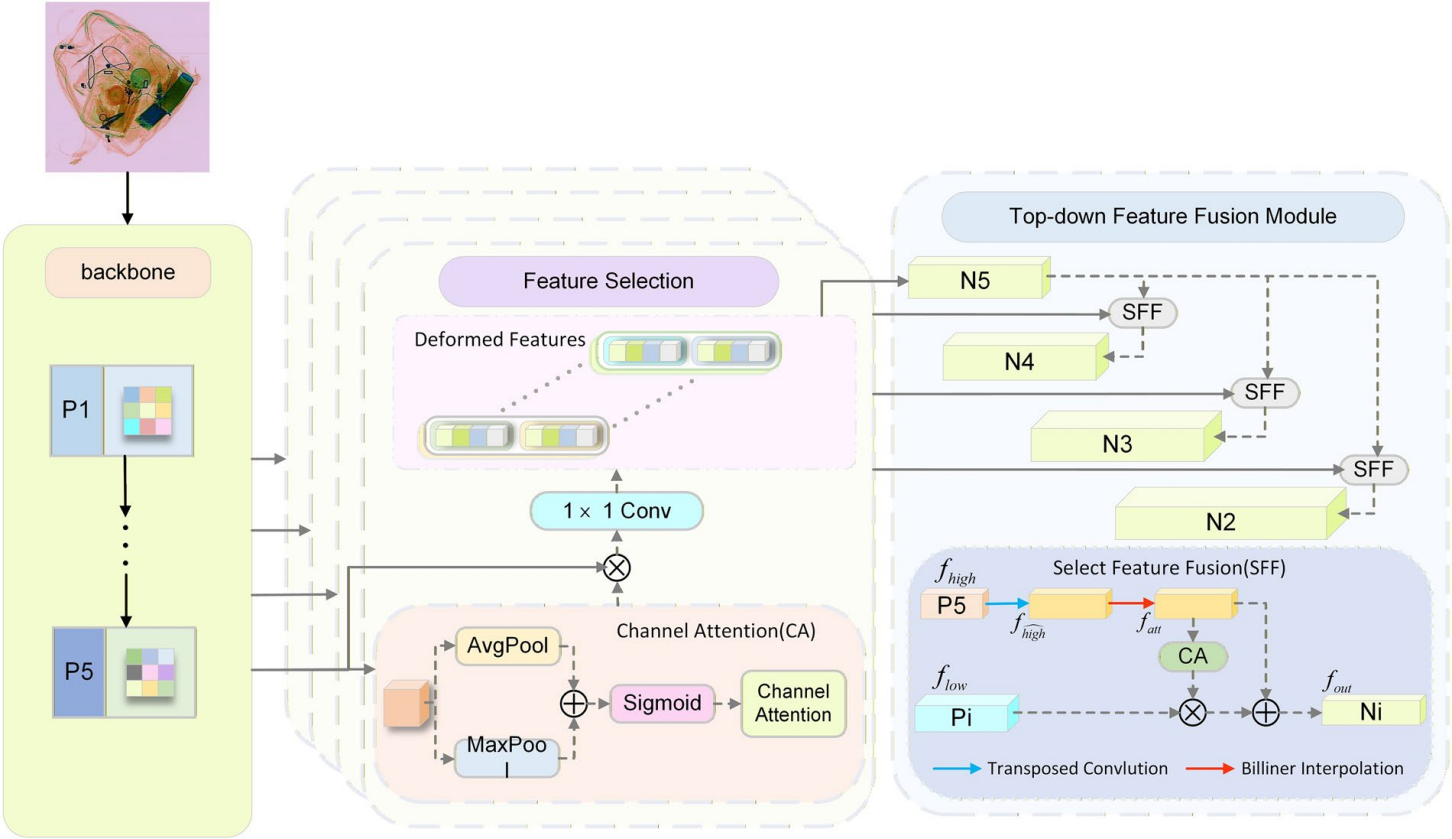
HSFPN network structure.

The High-level Screening-feature Pyramid Network (HSFPN)^37^ is a feature pyramid network specifically designed to address the multiscale problem, aiming to effectively fuse feature information across different scales. As shown in Fig. 10, the HSFPN architecture consists of two primary modules:(1) Feature Selection module, which filters feature maps across different scales to extract the most representative features; and (2) Feature Fusion module, which integrates high-level and low-level feature information through a selective fusion mechanism. Given an input high-level feature ${f}_{{high}}\in {R}^{C\times W\times H}$ and an input low-level feature ${f}_{{low}}\in {R}^{C\times {H}_{1}\times {W}_{1}}$, the high-level feature is initially expanded using a transposed convolution (T-Conv) with a stride size of 2 and a convolution kernel of 3 × 3, resulting in a feature size of ${f}_{\overline{{high}}}\in {R}^{C\times 2H\times 2W}$. Afterwards, to unify the dimensions of the high-level features and the low-level features, we use Bilinear Interpolation to either up-sample or down-sample the high-level features to obtain ${f}_{{att}}\in {R}^{C\times {H}_{1}\times {W}_{1}}\,$. The CA module is then employed to convert the high-level features into corresponding attention weights to filter the low-level features, upon obtaining features with consistent dimensions. Finally, the filtered lowlevel features are fused with the high-level features to enhance the model’s feature representation and obtain ${f}_{{out}}\in {R}^{C\times {H}_{1}\times {W}_{1}}$. The feature fusion process for feature selection is illustrated in Eqs. (10) and (11).

10 $${f}_{{att}}={BL}\left(T-{Conv}\left({f}_{{high}}\right)\right)$$

11 $${f}_{{out}}=\,{f}_{{low}}\ast {CA}({f}_{{att}})+{f}_{{att}}$$

where: ${f}_{{high}}\in {R}^{C\times H\times W}$, ${f}_{{low}}\in {R}^{C\times H\times W}$, $C$ denotes the number of channels, $H$ represents the height of the feature map, and $W$ indicates the width of the feature map.

To address challenges in practical security inspection scenarios—such as scale variations, complex backgrounds, and occlusions in X-ray images—this study introduces the CA-HSFPN (Coordinate Attention-based High-level Screening Feature Fusion Pyramid) module into the CSEC-Net model. This is achieved by replacing the original channel attention module in the HSFPN with the Coordinate Attention (CA)^38^ mechanism. By incorporating spatial coordinate information into feature maps, this enhancement enables the model to more effectively capture the precise locations and characteristics of contraband items.

Simultaneously, the enhanced model demonstrates improved capabilities in feature selection and fusion within the HSFPN. Crucially, the dimensionality reduction of feature maps significantly reduces the model’s parameter count, achieving lightweight model design while maintaining detection accuracy.

**Fig. 11:**
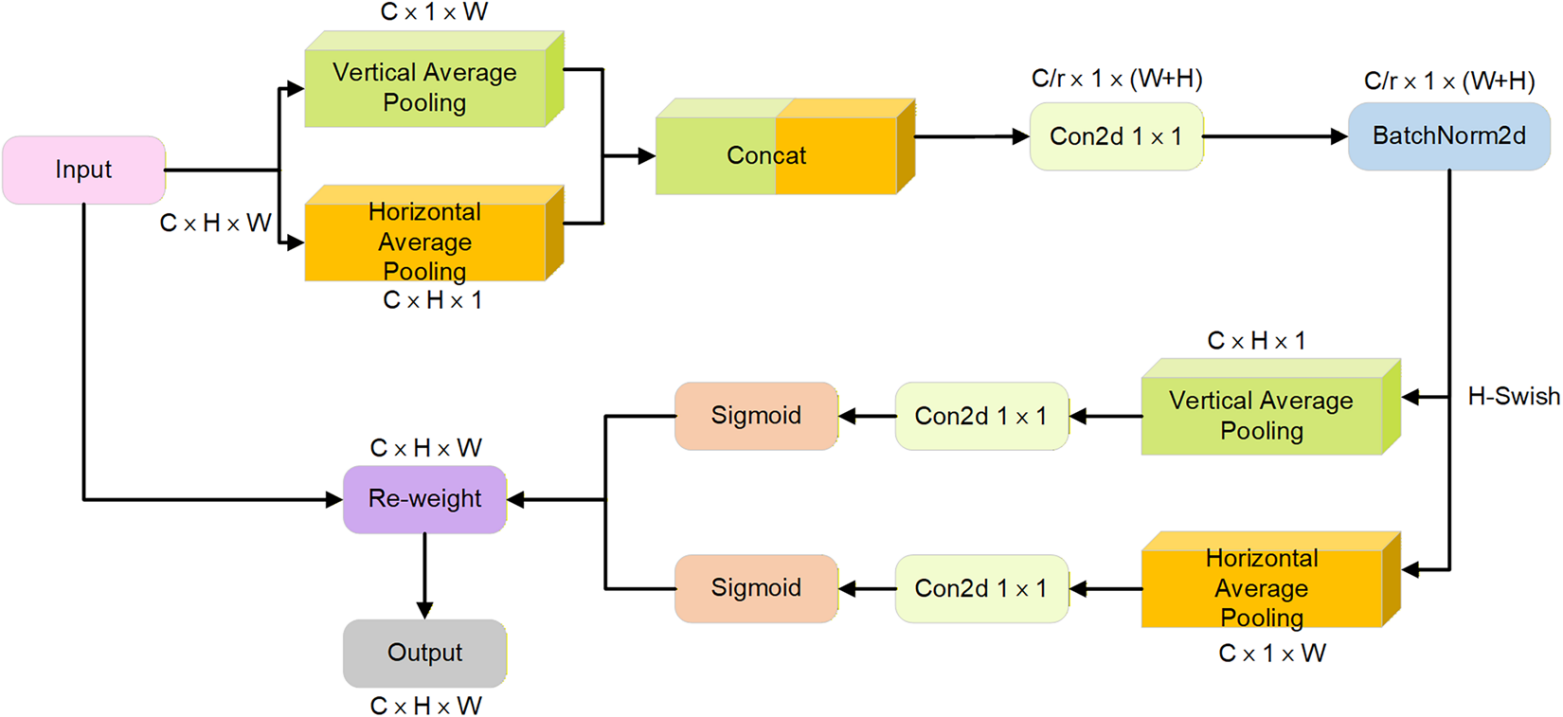
CA coordinate attention mechanism structure diagram.

Compared to channel attention and other complex attention mechanisms, Coordinate Attention adopts a lightweight design that integrates seamlessly into existing network architectures. This design substantially lowers model complexity and computational burden, making it highly suitable for real-world contraband detection applications and facilitating deployment in public settings. The structure of the Coordinate Attention module is illustrated in Fig. 11.

## Methods

### Input data

The SIXray^6^ dataset (https://github.com/MeioJane/SIXray) comprises 1,059,231 X-ray images, among which approximately 8,929 are positive samples containing prohibited items, and the remaining are negative samples without prohibited items. It covers 6 categories of common dangerous tool-type prohibited items, specifically including guns, knives, wrenches, pliers, scissors, and hammers. These items represent the most frequently intercepted mechanical threat objects in security inspection scenarios.

Developed under the leadership of the Institute of Computing Technology, Chinese Academy of Sciences (ICT CAS), the PIDray^9^ dataset (https://github.com/lutao2021/PIDray) achieves a core breakthrough by addressing the limitation of existing datasets—their “detachment from real-world security inspection pain points”. It is the first dataset to focus on the detection of intentionally concealed prohibited items. The PIDray dataset contains 47,677 high-quality X-ray images. Its test set is further subdivided into three subsets based on detection difficulty: “Easy” (prohibited items are unobstructed with clear outlines), “Hard” (prohibited items are partially occluded or confused with the background), and “Hidden” (prohibited items are intentionally concealed in clothing or sundries with highly blurred features).

### Data augmentation

**Fig. 12:**
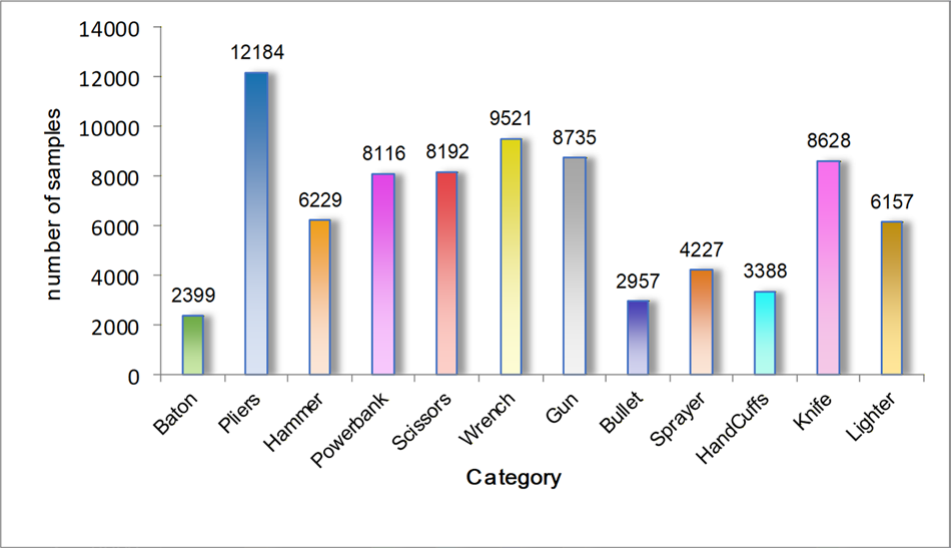
The class distribution of the synthesized dataset.

The experiments in this study were conducted on a balanced dataset created from two X-ray security inspection datasets, SIXray and PIDray, which have overlapping prohibited item categories. The SIXray dataset, comprising 8,929 samples, and the PIDray dataset, containing 56,606 samples, were integrated into a synthesized dataset. The class distribution of the synthesized dataset is illustrated in Fig. 12. Statistical analysis revealed a significant class imbalance issue: the *pliers*category had 12,184 instances, while *baton*contained only 2,399 samples. Such imbalance biases the model toward majority classes during training, leading to markedly reduced detection accuracy for minority classes and even missed detections.To address this, we propose a Class-Specific Augmentation Framework (CSAF), which selectively augments only images containing target minority-class objects. Crucially, annotations for non-target classes in these images are preserved to avoid distorting their features through excessive augmentation. Considering the grayscale characteristics of X-ray images and the specificity of prohibited item detection, we designed four physically meaningful transformations to enhance data diversity while preserving critical object features (e.g., contours and textures):

1. Horizontal Flip (p = 0.5): Simulates random object orientation in security screenings, mitigating model overfitting to directional biases.
2. Random Brightness/Contrast Adjustment (p = 0.4): Brightness range: [−15%, +15%], contrast range: [−20%, +20%], emulating X-ray imaging variations under different exposure settings.
3. Random Rotation (p = 0.6): Limited to [−15°, +15°] with bilinear interpolation for image resampling, ensuring bounding box precision post-rotation.
4. Gaussian Blurring (p = 0.3): Kernel size 3 × 3, simulating low-resolution X-ray noise to enhance model robustness.

**Fig. 13:**
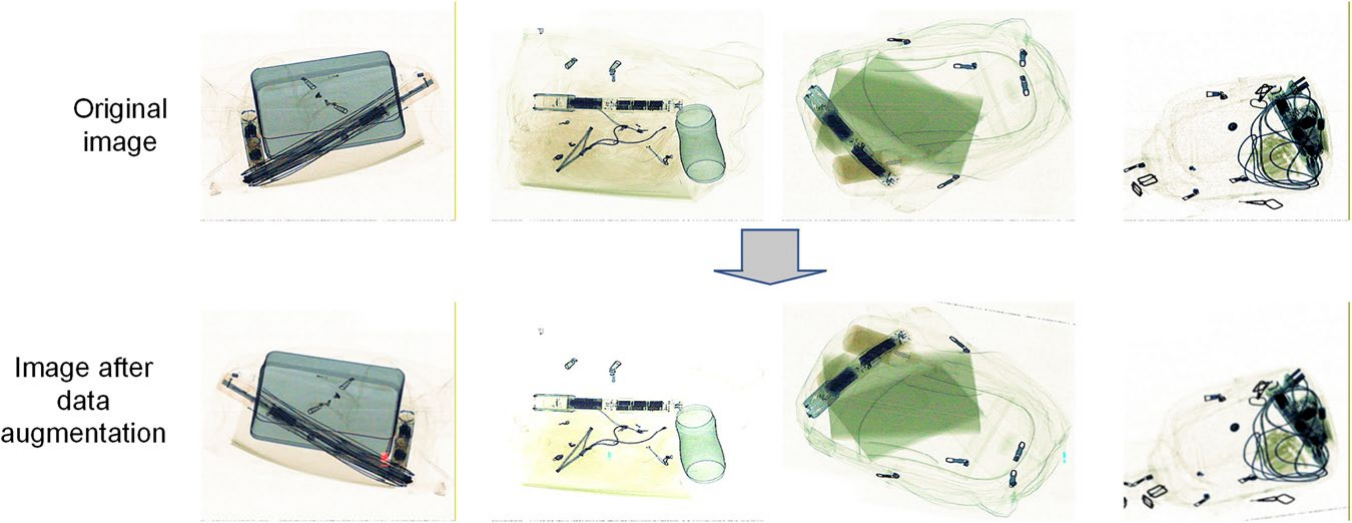
Augmented vs. Original Samples.

Bounding boxes followed the YOLO format (x_center, y_center, width, height, normalized values). To ensure label validity, a minimum visibility threshold of 0.4 was enforced: annotations were discarded if the post-augmentation visible area of a target bounding box fell below 40% of its original area, which prevents invalid labels from interfering with training. To avoid excessive sample redundancy, each image underwent at most one augmentation operation. The effect comparison of data augmentation is shown in Fig. 13.

### Data balancing

**Fig. 14:**
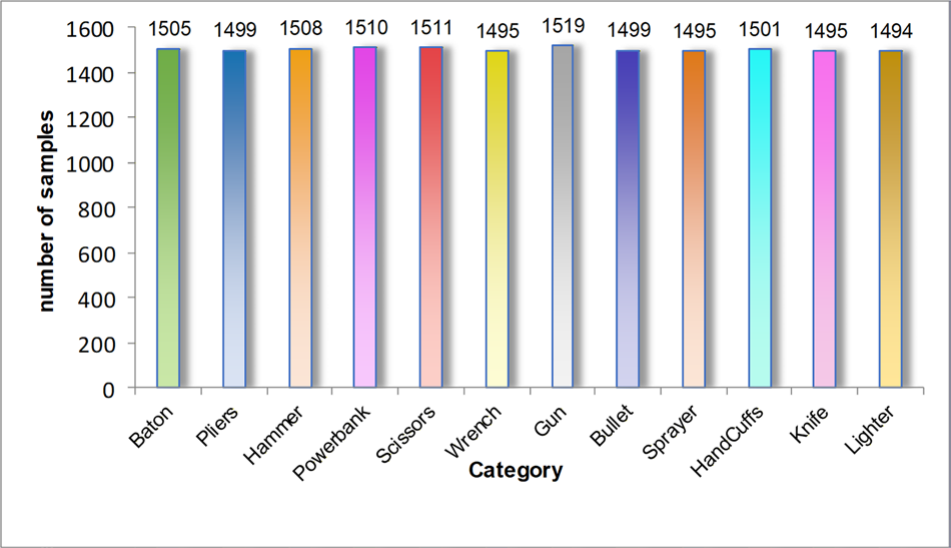
The number of sample cases corresponding to prohibited item categories.

Despite data augmentation, the dataset still exhibits class imbalance issues. To further achieve a balanced dataset while preserving data quality, this study employs the random undersampling method. The core principle of undersampling involves reducing the number of majority-class samples to equalize the distribution across all categories. Specifically, a subset of majority-class samples is randomly selected and retained to match the scale of minority-class samples, thereby constructing a class-balanced dataset. The target sample size for each class post-balancing is set at 1,500. For classes exceeding this threshold, random removal of samples is performed until their counts align with the target. As illustrated in Fig. 14, the final balanced dataset comprises 13,728 images, with all classes maintaining nearly identical sample sizes. This equilibrium provides an optimized foundation for subsequent model training, enhancing recognition capability across all categories and mitigating bias induced by data imbalance.

## Data Records

The balanced dataset is available on science data bank^39^. The dataset consists of 13,728 X - ray images, which are classified into 12 categories of contraband. The top - level directory of the dataset includes the “images” and “labels” directories. The “images” directory contains three sub - directories, namely “test”, “train”, and “val”, which store the images of the test set, training set, and validation set respectively, and the image files are in JPG format. The “labels” directory also contains corresponding “test”, “train”, and “val” directories, which store the annotation data of the test set, training set, and validation set respectively. These annotation data record the categories and positions of the contraband in the corresponding images, and the annotation files are in TXT format and use the YOLO format for annotation. In addition, a configuration file is stored in the dataset. This configuration file is used to explain the correspondence between different labels in the annotation data and the categories of contraband, as well as the path configuration of the dataset, and the format of the configuration file is YAML.

## Technical Validation

### Experimental environment and parameter settings

The experiments were conducted using NVIDIA A30 GPUs with 24GB of memory in a server environment, and the software environment included the the Red Hat Linux operating system (version 4.8.5). Python 3.9.23 and NVIDIA CUDA-11.6 were used for these experiments, with the coding environment facilitated by the PyTorch 1.12.1 deep learning framework.

The training process significantly impacts the model’s performance. Rational parameter configuration can accelerate model convergence and improve the accuracy of object detection. To ensure the fairness of the experiment, the same training strategy is applied to different networks. The parameters set during training are shown in Table 2, In the context of X - ray contraband detection, the selection of training hyperparameters plays a crucial role in determining the model’s performance. The initial learning rate () is set to 0.001, which is a moderately sized value. This moderate initial learning rate avoids the problem of unstable convergence caused by an excessively high learning rate in the early training stage, while also preventing the training process from being overly sluggish due to an overly low learning rate, thus enabling the model to start learning the features of contraband in X - ray images smoothly. The learning rate decay - related parameter enables an appropriate learning rate scheduling strategy. In the early stage of training, the model can utilize a relatively high learning rate to learn rapidly, and as training progresses, the learning rate gradually decreases. This allows the model to fine - tune its parameters more precisely, facilitating convergence to a better state and enhancing the precision of contraband detection in the later stage. With an epoch count of 200, the model has sufficient opportunities to learn the characteristic patterns of contraband in X - ray images. Considering that contraband may exhibit diverse manifestations in X - ray images in terms of shape and material, a sufficient number of training epochs helps the model fit the data more fully, reduces the risk of underfitting, and enables the model to better grasp the discrimination rules between contraband and non - contraband. The batch size is set to 16. On one hand, it can leverage the parallel computing capabilities of hardware such as GPUs to improve training efficiency compared to extremely small batches. On the other hand, unlike excessively large batches (such as hundreds), it avoids the problem that the gradient update of the model to the data may be too “rough” or requires excessive memory resources. For X - ray contraband detection, this batch size ensures training stability while allowing the model to calculate gradients based on a certain number of samples during each parameter update, making the learned features more generalizable and better adapted to X - ray contraband images in different scenarios. The weight decay coefficient is set to 0.0005. Combined with the AdamW optimizer (which itself introduces weight decay - based regularization), it further enhances the regularization effect. In the task of X - ray contraband detection, the model may learn some noise features or features useless for contraband identification in X - ray images. The weight decay mechanism can penalize the model’s weights, suppress the learning of these useless features, prevent the model from overfitting, and make the model focus more on learning the key features of contraband, thereby improving the model’s performance in detecting contraband in new, unseen X - ray images. The resulting dataset was partitioned into training, validation, and test sets according to an 8:1:1 ratio. In the experiments, all models were trained based on the specifications mentioned above, using this dataset with an input image size of 640 × 640.

**Table 2:** Training parameter settings.

parameter	setting	parameter	setting
epochs	200	lr0	0.001
batch size	16	lrf	0.01
optimizer	AdamW	warmup_momentum	0.8
imgsz	640	weight_decay	0.0005

### Evaluation metrics

To fairly and accurately assess the performance of the proposed method, the experiment employs the precision ($P$), recall ($R$), mean average precision (${mAP}$), model parameters (${parameters}$), floating-point operations (${FLOPs}$), and frames per second (${FPS}$). The metrics used in this paper are presented below:

12 $$P=\frac{{TP}}{{TP}+{FP}}\times 100 \% $$

where: ${TP}$ and ${FP}$ denote the number of true positive and false positive, respectively.

13 $$R=\frac{{TP}}{{TP}+{FN}}\times 100 \% $$

where: ${FN}$ represents the *count* of samples that are actually positive but incorrectly predicted as negative by the model.

14 $${AP}={\int }_{0}^{1}p\left(r\right){dr}\times 100 \% $$

15 $${mAP}=\frac{1}{N}\mathop{\sum }\limits_{k=1}^{k=N}{{AP}}_{k}\times 100 \% $$

where: $p(r)$ represents the P-R curve, N stands for the total number of categories and ${{AP}}_{k}$ indicates the $k$ th category value of ${AP}$.

16 $${FPS}=\frac{1}{{T}_{{pre}}+{T}_{{inference}}+{T}_{{post}}}$$

where: ${T}_{{pre}}$ encompasses image preparation operations including scaling, cropping, and normalization. ${T}_{{inference}}$ refers to the duration required by the model to analyze and recognize the input image. And ${T}_{{post}}$ covers result refinement steps such as parsing, filtering, and Non-Maximum Suppression (NMS).

In this experiment, mAP@50 quantifies the mean Average Precision at an IoU threshold of 0.5, where a predicted bounding box is deemed correct only if its overlap with the ground truth bounding box reaches or exceeds 50%. Similarly, mAP@50:95 denotes the mean Average Precision (mAP) computed by averaging AP values across ten distinct IoU thresholds from 0.50 to 0.95 with a step size of 0.05.

### Experiment on performance verification of balanced dataset

To verify the performance advantage of our balanced dataset over the original dataset under the same scale, we respectively conduct training and evaluation experiments on the contraband detection model of the proposed ASEA - Net for the two types of datasets. Firstly, 13,728 samples were randomly selected from the synthetic datasets of SIXray and PIDray as the experimental dataset, and the experimental results are shown in Tables 3, 4.

**Table 3:** Experimental results of ASEA-Net on synthesized dataset.

Class name	P/%	R/%	F1-Score/%	mAP@50/%	mAP@50:95/%
Baton	95.22	94.92	95.07	97.39	89.02
Plier	92.29	85.24	88.63	93.00	75.34
Hammer	96.45	97.06	96.75	98.56	92.76
Powerbank	90.32	94.34	92.28	96.14	78.91
Scissors	93.33	83.33	88.05	90.58	68.30
Wrench	93.93	84.85	89.16	92.63	78.23
Gun	94.89	91.47	93.15	96.22	76.08
Bullet	95.03	79.65	86.66	87.67	67.00
Sprayer	96.15	91.26	93.64	95.22	86.79
HandCuffs	94.74	98.70	96.68	99.18	89.20
Knife	93.37	77.88	84.93	86.29	64.15
Lighter	89.74	73.29	80.69	78.29	62.11
all	93.79	87.67	90.47	92.60	77.32

**Table 4:** Experimental results of ASEA-Net on balanced dataset.

Class name	P/%	R/%	F1-Score/%	mAP@50/%	mAP@50:95/%
Baton	97.99	93.35	95.61	98.09	81.81
Plier	88.78	84.21	86.43	90.26	70.70
Hammer	99.31	99.17	99.25	99.48	95.83
Powerbank	94.05	91.17	92.59	96.44	78.96
Scissors	95.42	81.11	87.68	88.05	67.83
Wrench	99.20	86.05	92.16	94.67	79.28
Gun	96.95	90.29	93.50	96.80	72.78
Bullet	97.36	87.74	92.30	94.70	80.41
Sprayer	98.78	95.33	97.03	98.81	91.16
Handcuffs	98.43	97.79	98.11	98.90	93.44
Knife	90.46	75.04	82.03	85.98	64.47
Lighter	92.67	76.25	83.66	88.41	62.95
all	95.78	88.17	91.72	93.55	78.30

From the perspective of single - class indicators, for most categories (such as Baton, Hammer, Gun, Handcuffs, etc.), the Precision, Recall, and F1 - Score of the model under the balanced dataset have been improved to varying degrees. Taking the Handcuffs category as an example, in the synthetic dataset, the Precision is 94.74%, the Recall is 98.70%, and the F1 - Score is 96.68%; under the balanced dataset, the Precision is increased to 98.43%, the Recall is 97.79%, and the F1 - Score reaches 98.11%. The improvements in precision and F1 - Score are quite significant, which indicates that the balanced dataset enables the model to identify targets of this category more accurately, while maintaining a good level in the ability to recall targets. Looking at the overall indicators, the average Precision of the synthetic dataset is 93.79%, the average Recall is 87.67%, and the average F1 - Score is 90.47%; the average Precision of the balanced dataset is increased to 95.78%, the average Recall is 88.17%, and the average F1 - Score is raised to 91.72%. As an indicator that comprehensively measures precision and recall, the improvement of the average F1 - Score reflects that the balanced dataset enables the model to achieve a better balance between accurately identifying and comprehensively recalling targets. In terms of mAP series indicators, the mAP@50 of the synthetic dataset is 92.60%, and the mAP@50:95 is 77.32%; the mAP@50 of the balanced dataset is increased to 93.55%, and the mAP@50:95 reaches 78.30%. The improvements of these indicators show that the model trained with the balanced dataset not only has higher detection accuracy under loose IoU thresholds but also has improved detection performance within stricter IoU threshold ranges, and the model has better accuracy in target localization and classification.

In conclusion, the balanced dataset shows better performance than the synthetic dataset in various evaluation indicators, effectively improving the accuracy, recall ability, and average detection accuracy of the target detection model under different IoU thresholds, which verifies the positive promoting effect of the balanced dataset on model performance.

### Comparative experiments on attention mechanism placement in the neck architecture

**Fig. 15:**
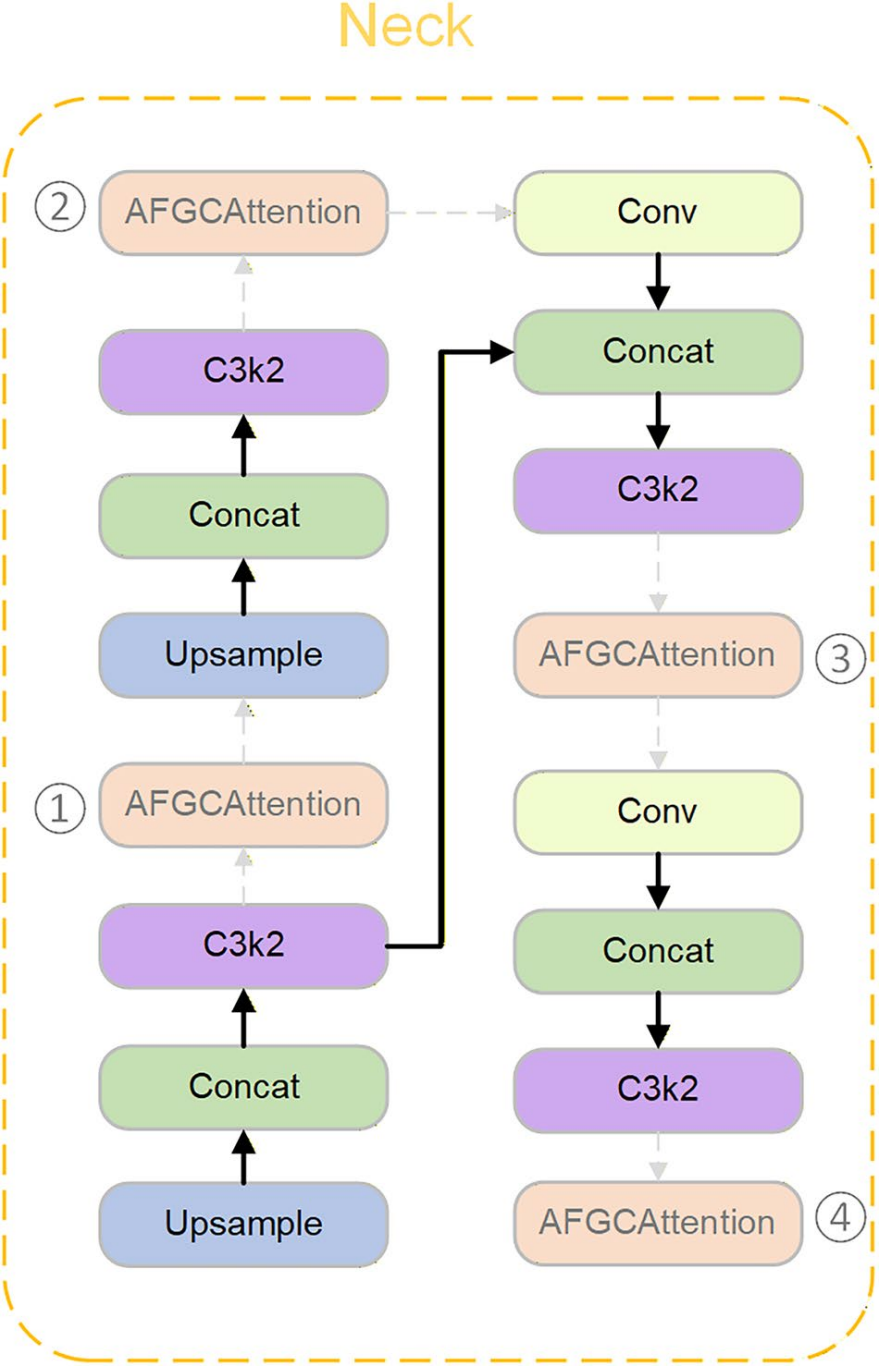
Position Diagram for Adding AFGCAttention module in the Neck.

To evaluate the impact of adding varying numbers of AFGCAttention modules at different positions in the model’s neck on algorithmic performance improvement, we conducted comparative experiments. As shown in Fig. 15, the neck of YOLOv11s contains four C3k2 modules. We sequentially added the AFGCAttention module after each of these four C3k2 modules for evaluation, followed by progressively increasing the number of attention modules in the experimental assessment.

The results presented in Table 5 demonstrate that adding the AFGCAttention module after the last C3k2 module in the neck maximizes the detection performance enhancement. Specifically, while maintaining unchanged GFLOPs and a 2.8% increase in parameters, the mAP@50 and mAP@50:95 improved by 0.36% and 0.26%, respectively, compared to the baseline model. However, introducing an excessive number of AFGCAttention modules into the network does not further improve model accuracy and may even degrade it. Therefore, based on experimental results, this paper integrates the AFGCAttention module after the final C3k2 module in the neck of the ASEA-Net model and directly connects it to the detection head, thereby enhancing overall model performance.

**Table 5:** Comparison experiment on attention mechanism placement in the neck architecture.

Models	mAP@50/%	mAP@50:95/%	Parameters/M	FLOPs/G	FPS
YOLOv11s	92.18	75.92	**9.41**	21.3	234
YOLOv11s + ①	91.93	75.89	9.48	21.3	225
YOLOv11s + ②	92.21	76.18	9.43	21.3	233
YOLOv11s + ③	91.80	75.46	9.48	21.3	230
YOLOv11s + ④	**92.54**	76.18	9.68	21.3	**236**
YOLOv11s + ③ + ④	92.22	75.44	9.74	21.3	227
YOLOv11s + ② + ③ + ④	91.99	75.79	9.76	21.3	227
YOLOv11s + ① + ② + ③ + ④	92.27	**76.21**	9.82	21.3	227

### Comparative experiments on different attention mechanisms

To further investigate the impact of various attention mechanisms within the neck network on model performance, we integrated different attention mechanisms at the identical position in the neck for comparative experiments. The experimental results are presented in Table 6. Benefiting from its ability to enhance fine-grained interactions between global and local information, thereby improving model performance, AFGCAttention achieved the highest mAP@50 of 92.54%. In contrast, the larger-parameter modules SDFM^40^ degraded model performance, while CAFM^41^ and ECA^42^ similarly reduced detection accuracy. The SimAM^43^ module yielded an mAP@50 equivalent to the baseline model, contributing minimally to performance improvement. Although MPCA^38^ attained the highest mAP@50:95 score of 76.33%, its parameter count increased by 14% compared to the baseline model. Overall, integrating AFGCAttention into the neck network delivers the optimal detection performance.

**Table 6:** Performance comparison of different attention mechanisms.

Models	mAP@50/%	mAP@50:95/%	Parameters/M	FLOPs/G	FPS
YOLOv11s	92.18	75.92	**9.41**	**21.3**	234
+CAFM^41^	91.92	75.70	10.63	22.3	222
+SimAM^43^	92.18	76.28	**9.41**	**21.3**	234
+TripletAttention^46^	92.3	**76.33**	**9.41**	**21.3**	227
+MPCA^38^	92.39	**76.33**	10.73	21.4	232
+MLCA^47^	92.27	75.81	**9.41**	**21.3**	233
+ECA^42^	91.94	75.96	9.42	**21.3**	228
+SDFM^40^	92.05	75.78	12.17	30.8	167
+AFGCAttention	**92.54**	76.18	9.68	**21.3**	**236**

### Comparative experiments on detection heads

To validate the advantages of the introduced SEAMHead detection module for prohibited item detection, we performed comparative experiments by replacing the detection head. The results are shown in Table 7. By incorporating the SEAMHead, the model’s ability to establish associations between occluded and non-occluded regions is strengthened, thereby improving detection accuracy. Experiments demonstrate that SEAMHead delivers the most significant performance improvement. While reducing the parameter count by 0.85%, it achieves mAP@50 and mAP@50:95 scores of 92.67% and 76.05% respectively, representing a substantial performance gain over the baseline model. Among the other detection modules tested, only MultiSEAM^32^, LSDECD^44^, and EfficientHead^45^ showed minor improvements compared to YOLOv11s. Notably, integrating MultiSEAM increased the model’s parameter count by 5.18 M. While LSDECD and EfficientHead are more lightweight than SEAMHead, they yielded only marginal accuracy improvements. In summary, the SEAMHead detection module, which effectively balances lightweight design and accuracy enhancement, demonstrates superior performance in detecting prohibited items within X-ray security inspection scenarios.

**Table 7:** Comparison experiment on detect head.

Models	mAP@50/%	mAP@50:95/%	Parameters/M	FLOPs/G	FPS
YOLOv11s	92.18	75.92	9.41	21.3	234
+LQE^48^	91.57	75.55	9.42	21.3	205
+MultiSEAM^32^	92.22	75.28	14.58	21.5	217
+RSCD^49^	89.24	66.97	9.66	22.0	**237**
+LSCSBD^50^	91.99	74.59	9.80	24.5	233
+LSDECD^44^	92.31	75.80	9.02	23.8	230
+EfficientHead^45^	92.15	75.99	**8.92**	**19.3**	232
+SEAM	**92.67**	**76.05**	9.33	20.8	222

### Ablation experiments

To validate the effectiveness of the proposed algorithmic improvements, we conducted ablation experiments using the original YOLOv11s network as the baseline. First, we performed ablation studies on our proposed ASEA-Net model. By incrementally integrating the EfficientNet_B0 feature extraction network, ADown downsampling module, SEAMHead detection head, and AFGCAttention mechanism, we could clearly observe the impact of each enhancement on model performance and computational overhead. The experimental results are summarized in Table 8. The data demonstrate that each improved module enhances the network’s detection performance. After replacing the backbone with EfficientNet_B0, while the FPS decreased to 132, the model’s parameter count reduced by 0.9 M, and both accuracy and mAP@50 increased by 0.94% and 0.79% compared to the baseline. This indicates that EfficientNet, at the cost of detection speed, not only reduces model parameters but also improves performance in detecting prohibited items in X-ray images.Further introducing the ADown downsampling module slightly reduced recall by 0.93%, while accuracy and FPS remained largely stable. However, mAP@50 and mAP@50:95 increased by 0.15% and 0.29%, respectively, and parameters decreased by an additional 0.54 M, effectively boosting model efficiency.Upon integrating the SEAMHead detection head, accuracy and mAP@50 further improved to 95.29% and 93.43%, although mAP@50:95 decreased by 0.5%. Additionally, the parameter count decreased to 7.9 M. Finally, adding the AFGCAttention mechanism to the neck increased parameters by 0.26 M but achieved optimal detection performance. Compared to the baseline, accuracy and mAP@50 improved by 1.46% and 1.33%, while overall parameters and FLOPs decreased by 13.3% and 27.7%, respectively. In summary, ASEA-Net outperforms the baseline YOLOv11s with higher accuracy and a more lightweight design, enabling more precise detection of prohibited items in X-ray images.

**Table 8:** Ablation study results for the ASEA-Net model.

EfficientNet	ADown	SEAMHead	AFCGAttention	P/%	R/%	mAP@50/%	mAP@50:95/%	Parameters/M	FLOPs/G	FPS
				94.32	86.44	92.18	75.92	9.42	21.3	**234**
✓				95.26	**88.32**	92.97	77.46	8.52	16.6	132
	✓			94.53	85.93	92.78	76.60	8.88	20.6	231
		✓		94.85	86.97	92.67	76.05	9.33	20.8	222
			✓	94.58	86.86	92.54	76.18	9.68	21.3	276
✓	✓			95.25	87.39	93.12	77.75	7.98	15.9	132
✓	✓	✓		95.29	88.31	93.43	77.25	**7.90**	**15.4**	128
✓	✓	✓	✓	**95.78**	88.17	**93.55**	**78.30**	8.16	**15.4**	128

Subsequently, we conducted an ablation study on our proposed lightweight CSEC-Net model by progressively integrating the CA-HSFPN module, EfficientNet_B0 feature extraction network, SEAMHead detection head, and C3k2-EMA module to observe the impact of these improvements on model performance and computational cost. The experimental results are summarized in Table 9. Experimental data indicate that among the four introduced improvement modules, all except the CA-HSFPN module enhance the detection performance. The introduction of CA-HSFPN reduced mAP@50 and mAP50:95 to 91.13% and 73.31%, respectively. However, it decreased model parameters by 2.79 M and FLOPs by 22.06%, demonstrating that the CA-HSFPN module significantly reduces model complexity with only minor performance degradation. Upon introducing the EfficientNet_B0 backbone, accuracy and mAP@50 increased to 95.72% and 92.73%, respectively. While outperforming the baseline in detection performance, the model’s parameters further decreased to 5.75 M, representing only 61.04% of the baseline’s parameter count. With the addition of the SEAMHead detection head, mAP@50 improved by 0.15%, parameters reduced to 5.66 M, and FLOPs decreased further to 12.7 G. Finally, integrating the C3k2-EMA module maintained parameter count and computational complexity unchanged, while mAP@50 rose to 93.05%. In summary, our lightweight CSEC-Net achieves a 39.91% reduction in parameters and a 40.38% reduction in FLOPs compared to YOLOv11s, while improving accuracy and mAP@50 by 1.24% and 0.87%, respectively. This delivers relatively ideal improvements at the cost of detection speed.

**Table 9:** Ablation study results for the CSEC-Net model.

CA-HSFPN	EfficientNet	SEAMHead	C3k2-EMA	P/%	R/%	mAP@50/%	mAP@50:95/%	Parameters/M	FLOPs/G	FPS
				94.32	86.44	92.18	75.92	9.42	21.3	**234**
✓				94.45	83.97	91.13	73.31	6.63	18.7	236
	✓			95.26	**88.32**	92.97	**77.46**	8.52	16.6	132
		✓		94.85	86.97	92.67	76.05	9.33	20.8	222
			✓	**96.05**	85.25	92.43	76.84	9.42	21.5	194
✓	✓			95.72	86.91	92.73	74.97	5.75	13.2	135
✓	✓	✓		94.17	88.28	92.88	75.45	**5.66**	**12.7**	132
✓	✓	✓	✓	95.56	87.58	**93.05**	75.35	**5.66**	**12.7**	130

### Comparative experiments

To evaluate the detection performance of our improved models and comprehensively validate the advantages of the proposed algorithm, we conducted comparative experiments against representative YOLO-series models and select RT-DETR-series models under identical datasets and training conditions. Experimental results in Table 10 demonstrate that our ASEA-Net achieves the highest mAP@50 at 93.55%. While YOLOv8n delivers the fastest detection speed and YOLOv11n maintains the smallest parameter count and lowest computational load, their insufficient detection accuracy renders them inadequate for public security screening applications. YOLOv11m achieves comparatively higher detection accuracy with 96.20% precision and 93.20% mAP@50, but its substantial parameter size and high computational load impose stringent hardware deployment requirements. Our CSEC-Net reduces parameters to only 28.24 percent of YOLOv11m’s count and decreases FLOPs by 50 G, requiring merely marginal sacrifices of 0.64 percentage points in precision and 0.05 percentage points in mAP@50. This significant reduction in computational resource consumption enables deployment on resource-constrained edge devices. Meanwhile, when compared to similarly-sized models such as YOLOv5s, YOLOv8s, and YOLOv10s, our ASEA-Net surpasses them across all accuracy metrics. RT-DETR-L and RT-DETR-ResNet50 not only exhibit higher parameter counts and computational loads, but also underperform in contraband detection accuracy within X-ray scenarios compared to our proposed ASEA-Net and CSEC-Net. These results collectively validate the feasibility and effectiveness of our algorithmic enhancements.

**Table 10:** Comparison of different detection algorithms on balanced datasets.

Models	P/%	R/%	mAP@50/%	mAP@50:95/%	Parameters/M	FLOPs/G	FPS
YOLOv3-tiny	90.66	79.74	87.50	65.82	9.53	14.3	364
YOLOv5s	94.84	85.16	91.91	74.78	7.82	18.8	247
YOLOv6s	91.96	83.01	89.55	72.70	15.98	42.8	245
YOLOv8n	92.37	84.54	90.22	72.44	2.69	6.8	**387**
YOLOv8s	95.03	85.15	91.97	76.54	9.83	23.4	243
YOLOv10s	92.81	84.52	91.49	75.42	7.22	21.4	248
YOLOv11n	91.76	82.59	89.85	71.54	**2.58**	**6.3**	339
YOLOv11s (baseline)	94.32	86.44	92.18	75.92	9.42	21.3	234
YOLOv11m	**96.20**	86.27	93.20	**79.01**	20.04	67.7	118
RT-DETR-L	93.19	**88.77**	92.13	78.66	28.47	100.7	59
RT-DETR-ResNet50	93.18	84.19	89.02	67.40	41.96	125.7	52
CSEC-Net (ours)	95.56	87.58	93.05	75.35	5.66	12.7	130
ASEA-Net (ours)	95.78	88.17	**93.55**	78.30	8.16	15.4	128

Table 11 presents a comparison between the proposed methods and other approaches on the PIDray dataset, with ∗ denotes methods specifically designed for contraband detection. Notably, the lightweight CSEA-Net developed in this study outperforms the vast majority of other models. Its overall mAP@50:95 across the three test sets is 0.7% higher than that of ForkNet, demonstrating superior detection performance with lower computational complexity. Furthermore, the designed ASEA-Net achieves the optimal detection performance: its overall mAP@50:95 is improved by 1.9% and 1.7% respectively compared to AEFNet-s^21^ and FDTNet^22^—the latest models under the same computational complexity. Specifically, the proposed ASEA-Net attains the highest mAP@50:95 values on the easy test set (78%) and hard test set (75.1%), representing significant improvements over other models. This validates the effectiveness of the proposed modifications for contraband detection tasks.

**Table 11:** Comparison of different detection algorithms on PIDray datasets.

Models	mAP@50:95/%			
	Easy	Hard	Hidden	Overall
RetinaNet^51^	66.4	58.1	45.8	56.8
Cascade-R-CNN^52^	69.3	62.8	48.0	60.0
Mask R-CNN^53^	70.7	63.5	49.6	61.3
SDANet^9^*	71.2	64.2	49.5	61.6
VFNet^54^	70.3	62.8	48.6	60.6
TOOD^55^	68.5	63.8	48.9	60.4
AEFNet-n^21^*	73.1	68.0	46.9	62.7
ATSS^56^	71.0	64.5	55.4	64.5
ForkNet^21^*	75.0	66.9	**58.6**	66.8
FDTNet^22^*	77.2	69.6	57.9	68.2
AEFNet-s^23^*	76.3	73.6	54.2	68.0
CSEC-Net(ours)	75.5	71.5	55.4	67.5
ASEA-Net(ours)	**78.0**	**75.1**	56.6	**69.9**

### Visualization experiments

**Fig. 16:**
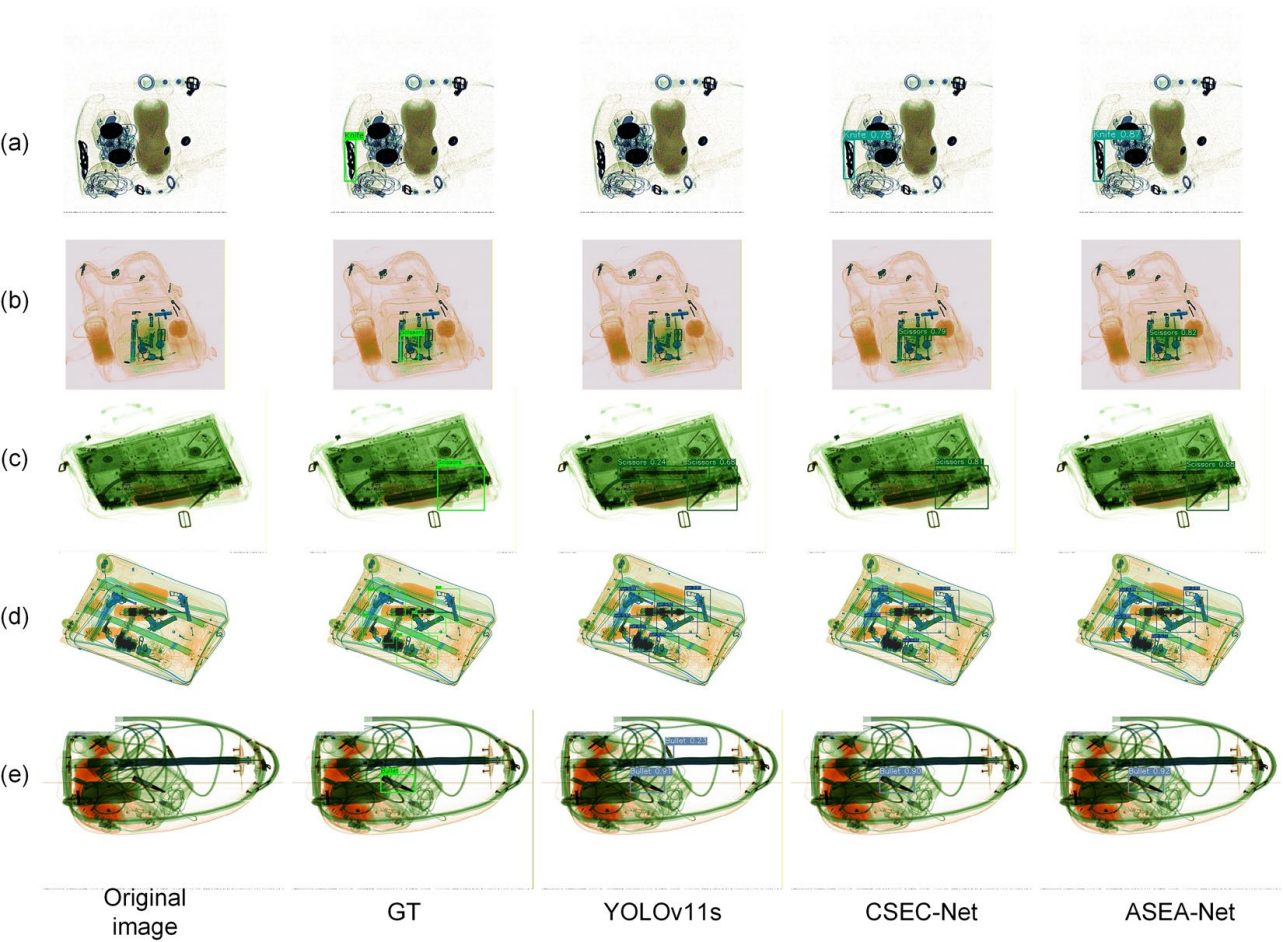
Comparison of detection results from different models for different sample types. Among these, (**a**) represents the non-occluded prohibited items scenario, while (**b**–**e**) represent overlapping and occluded prohibited items scenarios. The first column displays the original images, the second column shows the ground truth annotation, and the third column presents the detection results of the original YOLOv11s model. The fourth and fifth columns show the detection results of the CSEC-Net and ASEA-Net, respectively, proposed in this paper.

To demonstrate the superior performance of our proposed models in detecting prohibited items in X-ray images, we randomly selected several images depicting various types and quantities of prohibited items from the test set, including scenarios with overlapping and occlusion. Figure 16 illustrates the detection comparison among the baseline model YOLOv11s and our two improved models under different scenarios.

Comparing the detection performance of our improved models with YOLOv11s on the unobstructed scenario (a), the baseline model failed to detect the knife due to its small size and uncommon shape, whereas both our proposed ASEA-Net and CSEC-Net successfully identified the prohibited item. In scenarios with overlapping and occlusion, as shown in (b), the scissors were missed by the baseline model because occlusion rendered their contours indistinct; by contrast, our proposed methods accurately detected the occluded item.

In scenarios (c), (d), and (e), YOLOv11s exhibited varying degrees of false detections. Firstly, it misclassified an elongated object in (c) as scissors; secondly, it incorrectly identified a dark object in (d) as a gun; and finally, it falsely detected a data cable plug in (e) as a bullet. Conversely, our proposed models not only identify prohibited items more accurately across diverse complex scenarios but also yield higher confidence scores for detected items compared to the baseline model. Therefore, our proposed models demonstrate superior detection performance and accuracy for prohibited items in various scenarios.

**Fig. 17:**
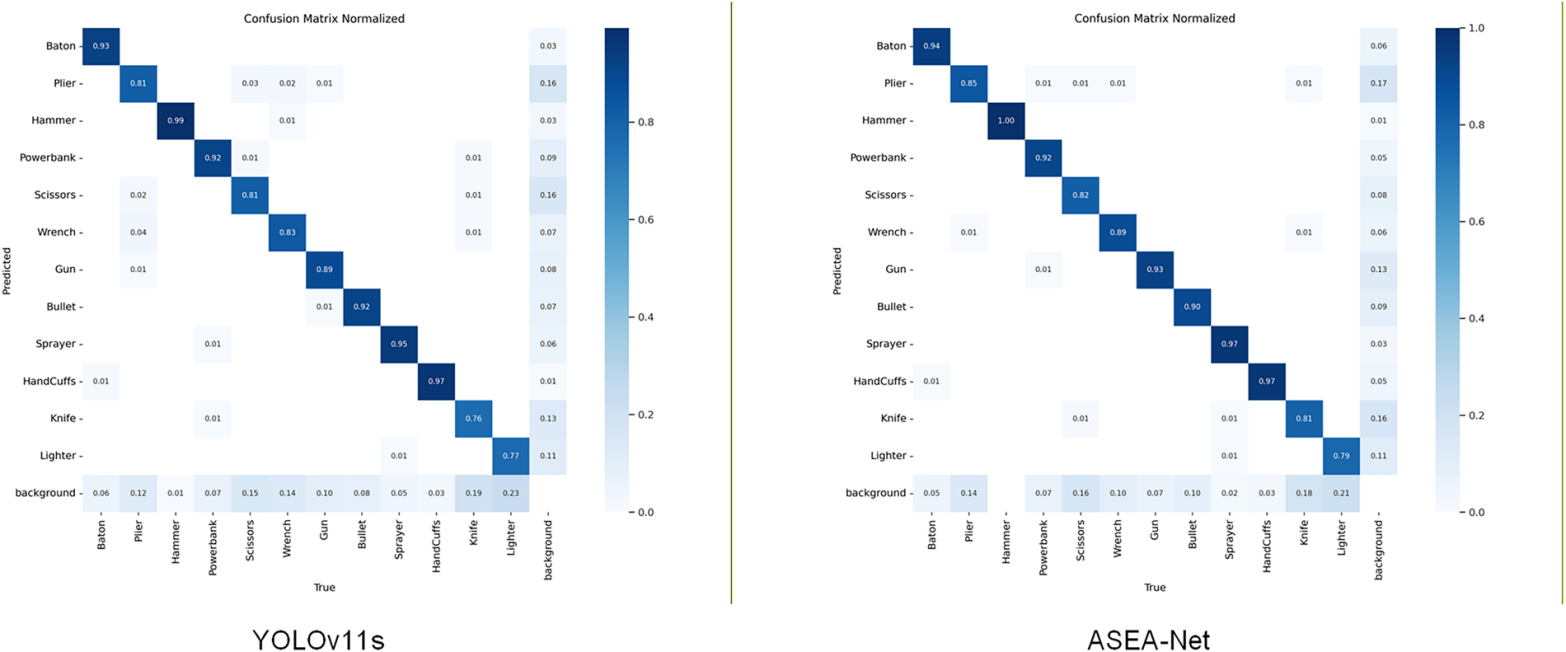
The confusion matrix normalized results of YOLOv11s and ASEA-Net.

**Fig. 18:**
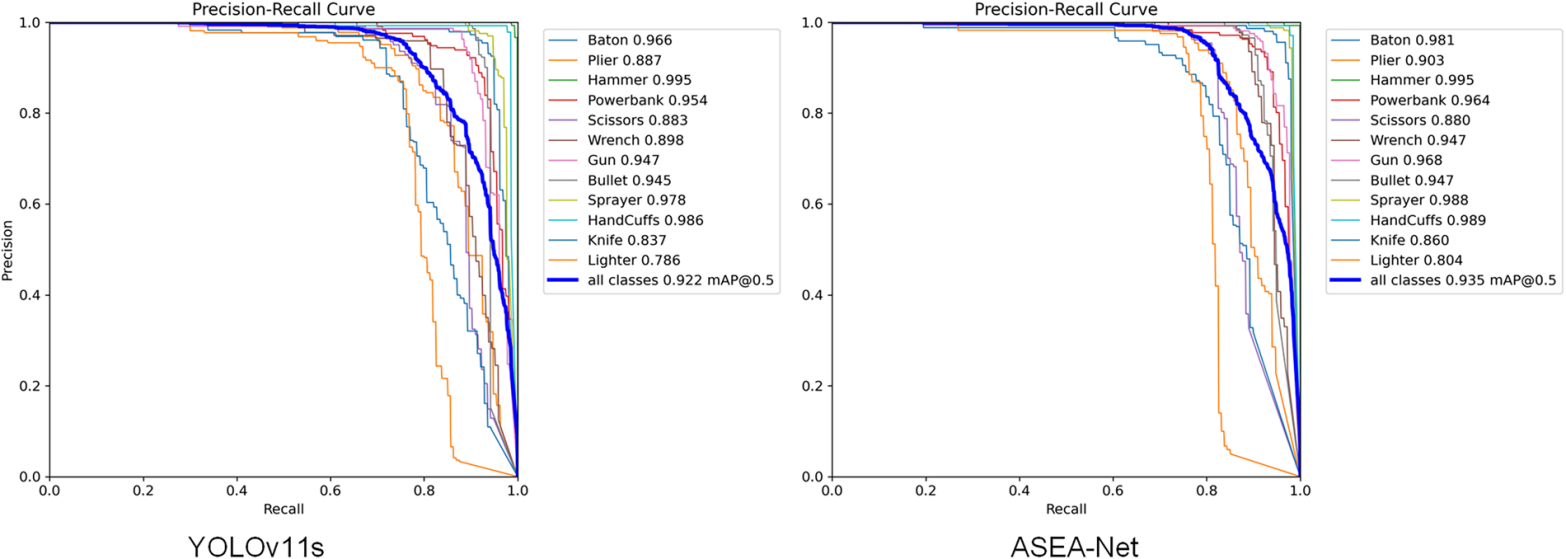
Comparison of PR curve.

To visualize detection accuracy across different prohibited item categories, we generated normalized confusion matrices comparing our improved ASEA-Net model against the baseline YOLOv11s, as presented in Fig. 17. The horizontal axis denotes true classes, while the vertical axis represents predicted classes, with background conventionally treated as an implicit category that does not impact actual model performance. Diagonal entries indicate the proportion of correct predictions per class, whereas off-diagonal entries reflect misclassifications. Our proposed ASEA-Net achieves higher detection accuracy than YOLOv11s for 9 prohibited item categories. The most significant improvement is observed for wrenches with a 6% accuracy increase. Only in the bullet category does ASEA-Net exhibit marginally lower accuracy compared to the baseline. These results demonstrate that our model enhances detection accuracy for diverse prohibited item types and delivers superior overall detection performance relative to the baseline model. We further generated Precision-Recall curves comparing ASEA-Net and YOLOv11s, as shown in Fig. 18. The left panel displays the PR curve for YOLOv11s, while the right panel shows ASEA-Net’s PR curve. Comparative analysis reveals that our proposed method achieves higher precision than the baseline model in 10 out of 12 prohibited item categories. The only exceptions are guns and scissors, where ASEA-Net exhibits marginally lower detection accuracy. Additionally, ASEA-Net improves the mAP@0.5 across all categories from 0.922 to 0.935, further attesting to the effectiveness of our proposed enhancements.

## Data Availability

The balanced X-ray security dataset^39^ is released at science data bank. The SIXray dataset is available at https://github.com/MeioJane/SIXray. The PIDray dataset is available at https://github.com/lutao2021/PIDray.

## Code availability

Some or all models, or code generated or used during the study are released at https://github.com/manba111/CSEC-Net.
